# STORM: spatial transcriptomics optimization by resolution via matrix factorization

**DOI:** 10.1093/bib/bbag324

**Published:** 2026-06-22

**Authors:** Deniz Gurarslan, Oscar Camargo, Omer Zeyveli, Yasin Almalioglu, Yanjun Li, Mehmet Turan, Tamer Kahveci

**Affiliations:** Institute of Data Science and Artificial Intelligence, Bogazici University, South Campus, Bebek, Besiktas, Istanbul 34342, Turkey; Department of Computer and Information Science and Engineering, University of Florida, 1889 Museum Road, Gainesville, FL 32611, United States; Department of Computer Engineering, Fatih Sultan Mehmet Vakif University, Halic Campus, Sutluce, Beyoglu, Istanbul 34445, Turkey; Department of Computer Science, University of Oxford, Wolfson Building, Parks Road, Oxford OX1 3QD, United Kingdom; Department of Computer and Information Science and Engineering, University of Florida, 1889 Museum Road, Gainesville, FL 32611, United States; Department of Medicinal Chemistry, Center for Natural Products, Drug Discovery and Development, University of Florida, 1345 Center Drive, Room P3-12, Gainesville, FL 32610, United States; Department of Computer Engineering, Bogazici University, Bebek, Besiktas, Istanbul 34342, Turkey; Department of Computer and Information Science and Engineering, University of Florida, 1889 Museum Road, Gainesville, FL 32611, United States

**Keywords:** spatial transcriptomics, multimodal data integration, tensor decomposition, biologically informed regularization, histology-guided modeling, gene–gene interaction networks, spatial gene–expression reconstruction, tumor microenvironment, tissue heterogeneity, computational pathology

## Abstract

Classic RNA sequencing dissociates cells from their native tissue architecture, discarding spatial information that critically shapes transcriptional programs in development, homeostasis, and cancer. However, current ST platforms often produce incomplete and noisy profiles due to technical limitations and tissue variability. These limitations obscure biologically meaningful spatial patterns and hinder downstream interpretation. Here, we introduce STORM (spatial transcriptomics optimization by resolution via matrix factorization), a machine learning framework that improves the fidelity of spatial transcriptomics data under severe sparsity. STORM formulates spatial transcriptomics recovery as a low-rank tensor decomposition problem and integrates multimodal biological priors through a principled regularization strategy. Specifically, the model jointly captures spatial continuity, tissue morphology derived from whole-slide histology images, and gene–gene interaction structure informed by protein–protein interaction networks. This method enables accurate reconstruction at unobserved locations while preserving biologically meaningful spatial structure. Across diverse lung tissue profiles, including both healthy and malignant samples, STORM consistently outperforms existing state-of-the-art methods in recovering spatial gene–expression patterns and remains robust even when a majority of spatial measurements are missing. By explicitly embedding biological structure into the reconstruction process, STORM provides a reliable foundation for high-resolution spatial transcriptomic analysis in settings where experimental data are sparse or incomplete. **Availability:** The source code developed in this study is publicly available at https://github.com/denizgurarslan/STORM.

## Introduction

Bulk and single-cell RNA sequencing quantify gene expression after dissociating cells from their native tissue architecture, thereby discarding spatial context, i.e. essential for understanding tissue organization and function [1]. Although these technologies provide precise transcript measurements, gene expression in intact tissues is strongly influenced by spatial location, local cell–cell interactions, and microenvironmental cues [2]. Loss of this context obscures cellular niches, spatial heterogeneity, and coordinated multicellular behavior.

Spatial transcriptomics (ST) preserves spatial coordinates of gene expression within intact tissue sections [1, 3], enabling direct analysis of how transcriptional programs organize in space. Each spatial spot corresponds to a physical tissue region and captures aggregated signals from one or more cells. This spatially resolved information is particularly important in oncology, where tumor progression, therapeutic response, and clinical outcomes depend on spatial heterogeneity and tumor–stroma interactions [4].

**Figure 1:**
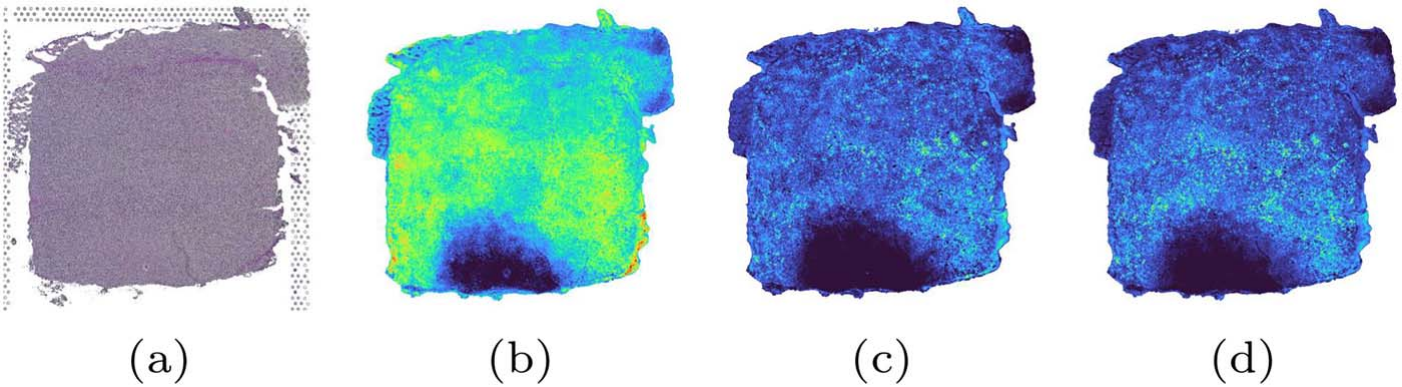
(a) WSI of a representative healthy brain tissue section. (b–d) ST heatmaps showing the expression levels of three genes associated with brain function and development: APOE, SYN1, and MEF2C. APOE is an Alzheimer’s disease-associated gene involved in lipid metabolism and neuronal signaling. SYN1 is linked to autism spectrum disorders and plays a central role in synaptic vesicle release. MEF2C regulates synaptic connectivity in the hippocampus and is associated with neurodegenerative disease.

An ST dataset integrates three complementary modalities: a high-resolution whole-slide image (WSI) that provides morphology, spatial coordinates that localize measurements, and a gene–expression matrix that quantifies transcription at each location . In standard ST pipelines, gene–expression measurements are inherently aligned with the corresponding WSI coordinates during data acquisition. Together, they support joint molecular and morphological analyses. Figure 1 illustrates a representative WSI and corresponding gene–expression heatmaps.

Current platforms fall into two categories. Imaging-based methods, such as Xenium [4], MERSCOPE [5], and CosMx [6], directly visualize transcripts to achieve single-cell or subcellular resolution. Sequencing-based platforms, including Visium [7], Stereo-seq [8], and GeoMx [9], use spatially indexed sequencing to provide genome-wide coverage. These approaches trade off spatial precision and molecular breadth: sequencing-based methods enable transcriptome-wide profiling but aggregate multiple cells per spot, blurring fine-scale structure [10], whereas higher resolution often increases sparsity and dropout [11]. Conversely, imaging-based methods offer high spatial fidelity but restrict measurements to predefined gene panels and require specialized, labor-intensive workflows [12]. Consequently, no platform simultaneously achieves high-resolution, whole-transcriptome coverage, and complete measurements.

Despite their promise, ST datasets frequently contain missing or unreliable signals caused by dropouts, spot bleed-over, limited sequencing depth, and variability in tissue quality. These artifacts obscure biologically meaningful spatial patterns and complicate downstream analysis, particularly in cancer studies that require accurate reconstruction of spatial gene–expression landscapes.

### Our contributions

We address a central limitation of ST: loss of biologically meaningful information due to sparse, noisy, or missing measurements. We introduce Spatial Transcriptomics Optimization by Resolution via Matrix-factorization (STORM), a machine learning framework that reconstructs high-fidelity spatial profiles under severe spot-level sparsity. STORM formulates recovery as a biologically informed tensor completion problem that integrates multimodal constraints. The model aligns spatial latent factors with histological morphology, enforces spatial smoothness across neighboring regions, and incorporates protein–protein interaction (PPI) priors to preserve functional gene relationships.

To account for heterogeneous measurement reliability, STORM employs a gene-aware weighted reconstruction objective that emphasizes genes with informative spatial variability while down-weighting weak or noisy signals. An adaptive loss-scaling strategy balances reconstruction and regularization terms, enabling stable optimization across datasets with different sparsity levels and resolutions.

Across diverse lung tissues spanning healthy and multiple cancer types, STORM consistently outperforms state-of-the-art methods, recovering accurate and spatially coherent expression patterns even when *∼ *70% of locations are missing. The resulting reconstructions respect tissue morphology, capture localized heterogeneity, and remain biologically interpretable, providing a general framework for ST analysis in studies of tissue organization and disease.

### Related works and gaps in the literature

A growing number of computational methods aim to improve the spatial resolution, completeness, and interpretability of ST data by exploiting auxiliary modalities or latent structure. Existing approaches largely differ in the primary information source they leverage, including histological images, spatial coordinates, or gene–expression statistics.

Image-guided methods incorporate tissue morphology to enhance spatial resolution. HisToGene integrates histological image patches with spatial positional embeddings using a modified Vision Transformer to generate super-resolution expression maps [13]. Similarly, iStar adopts a hierarchical Vision Transformer to model tissue structure across multiple scales and treats each gene’s spatial pattern as an image to refine resolution [14]. Although these approaches effectively exploit morphological cues, they rely predominantly on image features and typically treat genes independently, without explicitly modeling gene–gene dependencies or enforcing functional coherence in reconstructed profiles.

Coordinate-driven approaches instead rely solely on spatial geometry. STAGE employs a location-supervised autoencoder that aligns latent representations with spot coordinates and uses a sliced Wasserstein distance to regularize the spatial manifold, enabling interpolation at unmeasured locations [15]. However, STAGE ignores histological context and molecular interaction networks and restricts predictions to a subset of genes, limiting transcriptome-wide applicability.

Overall, most existing methods emphasize a single dominant modality—either histology or spatial coordinates—while modeling genes as independent variables. By neglecting joint modeling of spatial smoothness, tissue morphology, and gene–gene interactions, these approaches overlook key biological constraints and often degrade under high sparsity, complex tissue architecture, or heterogeneous cellular composition.

These limitations motivate a unified framework that integrates complementary modalities with explicit biological priors. STORM addresses this gap by jointly incorporating spatial continuity, morphological context, and gene interaction topology within a principled tensor decomposition formulation [13–15].

## Materials and methods

**Figure 2:**
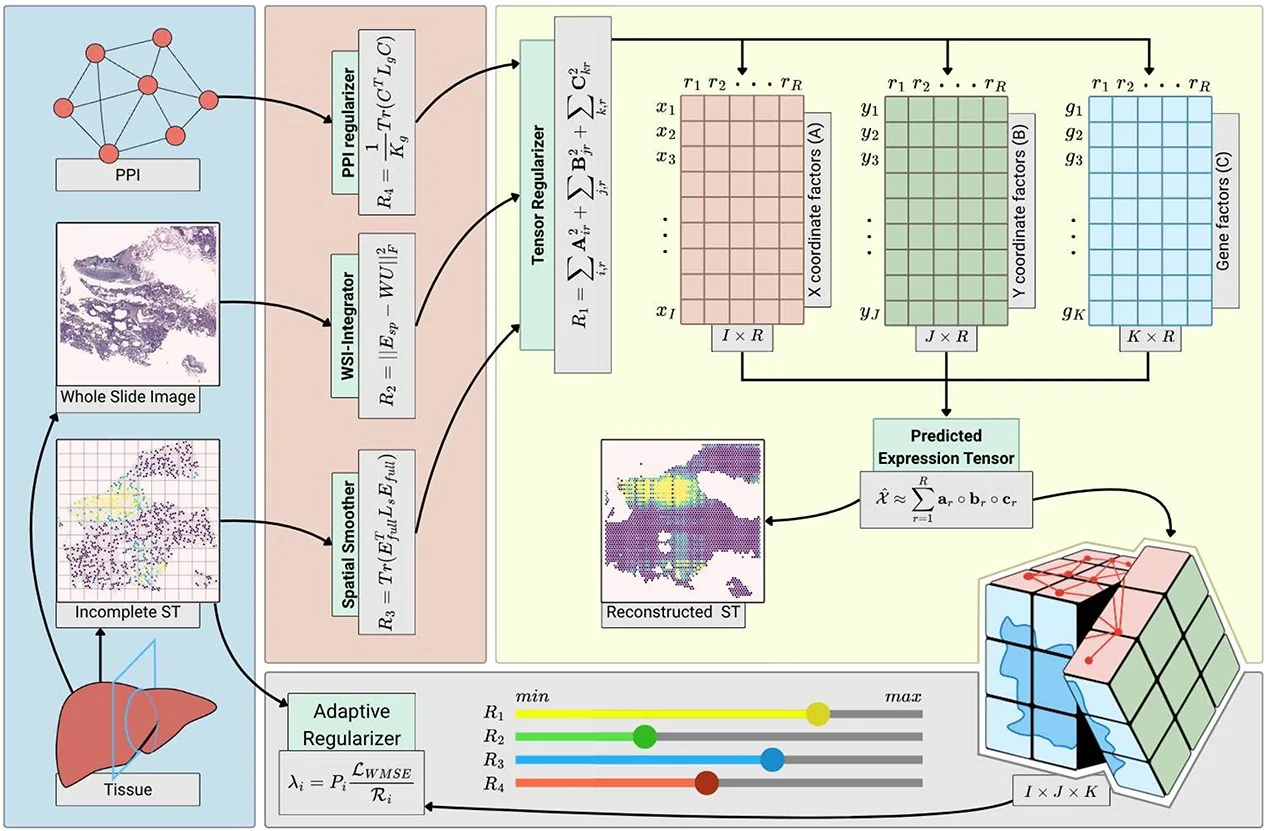
STORM: a multimodally regularized tensor decomposition framework for ST reconstruction. STORM formulates ST completion as a low-rank CP tensor decomposition of a sparse spatial *× * spatial *× * gene–expression tensor. Spatial and gene latent factors are jointly learned under four complementary regularization terms: $R_{1}$ magnitude regularization to prevent overfitting, $R_{2}$ alignment of spatial latent factors with tissue morphology using whole-slide histology image (WSI) embeddings, $R_{3}$ spatial smoothness enforced via a graph Laplacian to propagate information across neighboring tissue locations, and $R_{4}$ biological consistency imposed through gene–gene interaction constraints derived from PPI networks. Together, these multimodal constraints enable robust reconstruction of missing spatial transcriptomic measurements while preserving spatial structure, tissue morphology, and functional gene relationships.

In this section, we introduce STORM, a unified computational framework for reconstructing ST profiles at unobserved or unreliable spatial locations. STORM is designed to address the intrinsic sparsity and noise characteristics of spatial transcriptomic data while preserving biologically meaningful spatial and molecular structures. At its core, STORM formulates ST recovery as a low-rank tensor decomposition problem, in which spatial coordinates and gene–expression measurements are jointly modeled within a compact latent representation. This formulation enables the model to exploit shared structure across spatial dimensions and genes, thereby facilitating information propagation from observed locations to missing regions. STORM extends classical tensor decomposition by incorporating biologically informed regularization terms that explicitly encode key principles governing transcriptional organization in tissues. Specifically, the framework integrates complementary sources of biological and spatial information through regularization components that enforce consistency with tissue morphology derived from whole-slide histology images, promote smooth transcriptional transitions across neighboring spatial locations, and preserve functional relationships among genes based on known gene–gene interaction networks. By embedding these multimodal biological priors directly into the optimization objective, STORM ensures that reconstructed expression profiles remain coherent with underlying tissue architecture and molecular interaction structure, rather than reflecting purely statistical interpolation. Through this combination of low-rank modeling and biologically grounded regularization, STORM provides a principled and flexible approach for ST reconstruction, i.e. robust to severe data sparsity and heterogeneous noise. Figure 2 illustrates the STORM framework. In Supplementary Material SM1, we formally define the problem setting, introduce the tensor decomposition formulation, and describe each regularization component in detail.

## Results

To comprehensively assess the effectiveness of STORM, we designed a systematic experimental evaluation that examined reconstruction accuracy, robustness to missing data, and biological plausibility across diverse tissue contexts. Our evaluation strategy is guided by three central questions: (i) to what extent can STORM accurately recover missing spatial gene–expression values under varying degrees of sparsity, (ii) how does its performance compare with state-of-the-art spatial transcriptomic reconstruction methods, and (iii) whether the reconstructed expression patterns preserve biologically meaningful spatial and functional structures? To this end, we conducted experiments across multiple tissue types, masking regimes, and gene subsets, and evaluated the performance using both global correlation-based metrics and localized spatial error analyses. Importantly, all evaluations were performed on real human ST datasets paired with histological images and gene interaction networks, ensuring that conclusions reflect realistic experimental conditions rather than synthetic benchmarks. To further assess robustness under noisy observations, we additionally conducted a sensitivity analysis based on controlled noise perturbations (see Supplementary Material, Section SM4).

### Datasets

We evaluated the proposed method using publicly available ST datasets. In the following section, we describe the datasets used in the experiments.

#### Spatial transcriptomics data

 We trained and evaluated STORM using three ST datasets: HEST-1k [16], HER2ST [17], and spatialLIBD [18]. From HEST-1k, we selected eight lung tissue profiles, comprising four healthy samples from the Mendeley (MEND) collection (MEND41, MEND47, MEND89, and MEND90) and four cancer samples from the 10*× * Genomics (TENX) collection (TENX62, TENX72, TENX118, and TENX141). These cancer profiles included lung squamous cell carcinoma (LUSC), lung neuroendocrine tumor (LNET), and lung adenocarcinoma (LUAD) samples. Among the HEST-1k profiles, TENX118 and TENX141 were generated using the imaging-based Xenium platform, whereas the remaining six profiles were acquired using the sequencing-based Visium platform. In addition, we included four HER2ST breast cancer profiles (A1, E1, F1, and H1) and four spatialLIBD human brain profiles (151 507, 151 510, 151 669, and 151 673). Thus, the full benchmark comprised 16 tissue profiles in total. To assess robustness under varying levels of data sparsity, we generated masked versions of each ST profile by hiding transcription values at a specified fraction of spatial locations. We considered masking rates of 30%, 50%, and 70%, representing increasing levels of missing data. Masks were constructed incrementally to preserve the spatial substructure: the set of hidden spots at 30% is a subset of that at 50%, and the 50% mask is a subset of the 70% mask. For each masking rate, we generated three independent masked realizations by randomly selecting spatial locations to be hidden. In total, this procedure yielded 144 profiles for evaluation (16 tissues *× * 3 masking rates *× * 3 repeats).

#### Network data

We obtained human PPI networks from the STRING database [19], which provides both experimentally validated and computationally inferred interactions representing physical binding and functional associations between genes. Each interaction is associated with a confidence score in the range *[0,1]*, with higher values indicating stronger supporting evidence. We apply a minimum confidence threshold of 0.65 to retain high-confidence interactions. After filtering, the resulting PPI network contained 16 718 genes and 593 158 gene–gene interactions. For each ST profile, we further restricted the network to interactions involving the genes present in the corresponding dataset.

#### Competing methods

We compared STORM with four recent baselines: iStar [14], STAGE [15], Nicheformer [20], and Novae [21]. To ensure a fair and transparent comparison across methods, all models were evaluated on the same held-out split, using the same target gene set and the same Pearson-based evaluation protocol. Notably, iStar does not directly output spot-level gene–expression values, but instead generates super-resolution expression maps on a regular pixel grid for each gene. To make iStar comparable with the other methods, we converted these maps into spot-level predictions by aggregating the predicted intensities of super-resolution pixels within each spot. For Novae, which does not natively output reconstructed expression matrices, we adapted its pretrained representations to produce directly comparable spot-level expression predictions.

#### Benchmarking against state-of-the-art methods

To rigorously assess the performance of STORM, we benchmarked it against four recent methods for ST recovery. Specifically, we compared STORM to both dedicated reconstruction methods and adapted foundation-model baselines, thereby covering the main types of approaches considered in this study. To ensure a fair and transparent comparison, all methods were evaluated on the same held-out split, using the same target gene set and the same Pearson-based evaluation protocol. Our evaluation was designed to probe not only overall reconstruction accuracy but also robustness across various biological contexts and levels of spatial sparsity. We considered both healthy and malignant lung tissue profiles, as well as multiple masking ratios, to reflect realistic experimental conditions encountered in ST studies. Prediction accuracy was quantified using Pearson’s correlation (PC) between reconstructed and ground-truth expression values at held-out spatial locations. We report the results at two complementary resolutions: an aggregate analysis summarizing performance across profiles and experimental repeats, followed by a detailed per-sample evaluation that highlights tissue-specific behavior and failure modes. Together, these analyses provide a comprehensive and biologically grounded assessment of STORM relative to the competing baselines.

#### Aggregate analysis

**Figure 3:**
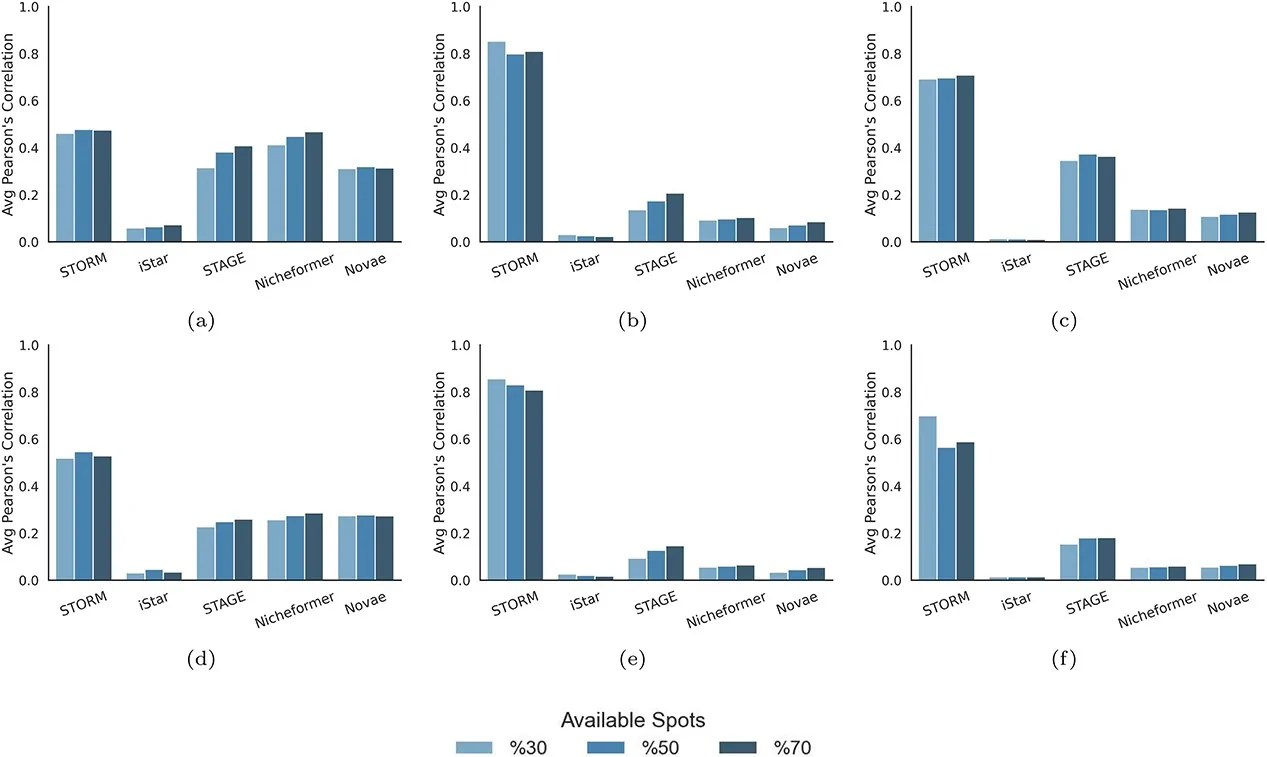
Robust recovery of biologically meaningful spatial transcriptional structure. Prediction accuracy quantified by PC for five methods: STORM, iStar, STAGE, Nicheformer, and Novae—across ST datasets under increasing levels of spot masking (30%, 50%, and 70%). Panels show results for (a) HEST-1k with the top 500 genes, (b) her2st with the top 500 genes, (c) spatialLIBD with the top 500 genes, (d) HEST-1k with the top 10 000 genes, (e) her2st with the top 10 000 genes, and (f) spatialLIBD with the top 10 000 genes. Across all masking regimes, tissue types, and gene set sizes, STORM consistently achieves higher correlation with ground-truth expression, indicating superior preservation of spatially resolved transcriptional patterns under severe data sparsity. Notably, the performance advantage persists in both healthy and malignant tissues, suggesting that STORM captures biologically structured variation rather than overfitting dataset-specific artifacts. Pearson correlation values are averaged over three independent random masking trials.

Our first experiment compares the predictive accuracy of STORM against the existing methods, iStar, STAGE, Nicheformer, and Novae, using PC between the predicted transcription values at masked spatial locations and their corresponding ground-truth values. Each method was evaluated at masking rates of 30%, 50%, and 70% for each tissue profile. For every masking level, the experiment was repeated three times using independently sampled random masks and the reported PC values were averaged across all repeats. The aggregate results are shown in Fig. 3. Across majority of experimental settings, STORM consistently achieved substantially higher PC values than competing methods. This performance advantage is observed for both healthy and cancerous tissues, across low and high masking rates, and for gene subsets ranging from highly variable genes to large collections that include genes with diverse transcriptional variability. These results highlight not only the overall superiority of STORM in reconstructing missing spatial transcriptomic data but also its robustness to heterogeneous dataset characteristics. In contrast, the competing methods showed substantially less consistent behavior across datasets and experimental settings. iStar generally yielded the lowest PC values, whereas STAGE improved upon iStar but remained clearly below STORM in the aggregate comparisons. Nicheformer and Novae were more competitive in a subset of profiles, particularly within HEST-1k and under the 500-gene setting, and occasionally achieved the best score for individual samples. However, these gains were not consistent across datasets and masking ratios, and did not translate into higher dataset-level averages. By contrast, STORM achieved the highest average PC in every dataset, gene-set, and masking-level combination considered in this benchmark. Although the effect of masking ratio was not strictly monotonic across all datasets, STORM remained the most accurate method overall under both sparse and less sparse observation regimes. These findings indicate that STORM combines strong reconstruction accuracy with stable cross-dataset performance, whereas the competing methods are either substantially weaker or more dependent on the specific dataset and evaluation setting.

#### Detailed analysis

Next, we examined the prediction accuracy of all compared methods at the level of individual tissue profiles. For each masking rate and method, we reported the average PC across three independently generated random masks. The experiments were conducted using both the top 500 and 10 000 highly variable genes. The detailed results are presented in Table 1. Across 72 out of 96 combinations of tissue profile, masking ratio, and gene set size (i.e. 75% of the test cases), STORM achieved the highest PC values. Moreover, the performance gap between STORM and the competing methods is consistently substantial. Specifically, in 71 of the 72 cases, the difference between STORM and the next best method exceeded 0.1, and in 64 cases the gap exceeded 0.2. Only 23 cases were observed in which a competing method outperformed STORM; among them, Nicheformer, Novae, and STAGE achieved the highest PC values in 15, 6, and 2 cases, respectively. In one additional case, STORM and Nicheformer achieved identical PC values. In contrast, iStar yielded consistently low PC values across all experimental settings. We attribute this behavior to the fact that iStar is not explicitly designed for spot-level reconstruction of ST data and, therefore, does not fully account for the structural and statistical properties of ST measurements.

**Table 1:** Comprehensive comparison of reconstruction accuracy across datasets, sparsity levels, and gene sets, showing PC values for five methods (M1 = STORM, M2 = iStar, M3 = STAGE, M4 = Nicheformer, and M5 = Novae) evaluated on ST profiles from the HEST-1k, her2st, and spatialLIBD datasets under increasing levels of spot masking (30%, 50%, and 70%)

**Profiles**	**HEST-1k: 10 000 Genes**															**NoG**
	**70% Downsampled**					**50% Downsampled**					**30% Downsampled**					
	**M1**	**M2**	**M3**	**M4**	**M5**	**M1**	**M2**	**M3**	**M4**	**M5**	**M1**	**M2**	**M3**	**M4**	**M5**	
MEND41	**0.60**	0.02	0.10	0.08	0.17	**0.70**	0.02	0.14	0.09	0.22	**0.61**	0.01	0.17	0.09	0.20	33 538
MEND47	**0.55**	0.02	0.11	0.07	0.17	**0.68**	0.02	0.13	0.08	0.17	**0.60**	0.01	0.16	0.09	0.17	33 538
MEND89	**0.50**	0.14	0.21	0.12	0.25	**0.51**	0.17	0.24	0.13	0.24	**0.51**	0.16	0.25	0.14	0.23	17 943
MEND90	**0.54**	0.04	0.17	0.15	0.20	**0.55**	0.06	0.21	0.17	0.21	**0.57**	0.05	0.25	0.18	0.21	17 943
TENX62	**0.47**	0.06	0.29	0.32	0.23	**0.45**	0.07	0.32	0.34	0.20	**0.45**	0.04	0.34	0.35	0.18	18 085
TENX72	**0.50**	−0.02	0.34	0.30	0.18	**0.51**	0.01	0.35	0.32	0.19	**0.50**	0.01	0.37	0.32	0.19	18 085
TENX118	0.47	0.00	0.19	0.43	**0.51**	0.47	0.02	0.18	0.46	**0.51**	0.47	0.00	0.32	0.48	**0.50**	541
TENX141	0.50	−0.01	0.40	**0.58**	0.48	0.51	0.02	0.42	**0.62**	0.48	0.51	0.00	0.23	**0.64**	0.49	541
**Average**	**0.52**	0.03	0.23	0.26	0.27	**0.55**	0.05	0.25	0.27	0.28	**0.53**	0.03	0.26	0.29	0.27	17 526
**Profiles**	**HEST-1k: 500 Genes**															**NoG**
	**70% Downsampled**					**50% Downsampled**					**30% Downsampled**					
	**M1**	**M2**	**M3**	**M4**	**M5**	**M1**	**M2**	**M3**	**M4**	**M5**	**M1**	**M2**	**M3**	**M4**	**M5**	
MEND41	**0.62**	0.01	0.19	0.15	0.17	**0.66**	0.01	0.24	0.17	0.20	**0.59**	0.01	0.26	0.16	0.16	33 538
MEND47	**0.70**	0.07	0.19	0.15	0.17	**0.71**	0.05	0.27	0.17	0.17	**0.55**	0.05	0.25	0.20	0.16	33 538
MEND89	0.30	0.18	0.27	**0.36**	0.22	0.32	0.19	0.33	**0.40**	0.21	**0.53**	0.25	0.35	0.42	0.20	17 943
MEND90	**0.50**	0.10	0.27	**0.50**	0.22	0.52	0.14	0.33	**0.54**	0.23	0.53	0.15	0.37	**0.57**	0.24	17 943
TENX62	0.33	0.09	0.40	**0.55**	0.26	0.31	0.10	0.46	**0.59**	0.26	0.33	0.10	0.51	**0.63**	0.25	18 085
TENX72	0.26	0.05	0.60	**0.61**	0.47	0.32	0.05	0.59	**0.66**	0.51	0.28	0.06	**0.70**	0.68	0.52	18 085
TENX118	0.47	−0.17	0.48	0.42	**0.51**	0.47	−0.18	0.41	0.44	**0.51**	0.47	−0.18	0.44	0.46	**0.50**	541
TENX141	0.51	0.14	0.15	**0.57**	0.47	0.51	0.14	0.44	**0.61**	0.48	0.52	0.14	0.39	**0.63**	0.49	541
**Average**	**0.46**	0.06	0.32	0.41	0.31	**0.48**	0.06	0.38	0.45	0.32	**0.48**	0.07	0.41	0.47	0.31	17 526
**Profiles**	**HER2ST: 10 000 Genes**															**NoG**
	**70% Downsampled**					**50% Downsampled**					**30% Downsampled**					
	**M1**	**M2**	**M3**	**M4**	**M5**	**M1**	**M2**	**M3**	**M4**	**M5**	**M1**	**M2**	**M3**	**M4**	**M5**	
A1	**0.85**	0.02	0.10	0.04	0.03	**0.82**	0.01	0.14	0.05	0.04	**0.81**	0.01	0.16	0.05	0.04	15 045
E1	**0.86**	0.02	0.09	0.07	0.05	**0.85**	0.02	0.13	0.07	0.06	**0.77**	0.02	0.14	0.07	0.07	15 701
F1	**0.83**	0.03	0.07	0.03	0.02	**0.81**	0.03	0.10	0.03	0.02	**0.80**	0.02	0.12	0.03	0.03	14 861
H1	**0.88**	0.04	0.11	0.08	0.04	**0.85**	0.03	0.14	0.09	0.06	**0.85**	0.02	0.17	0.10	0.08	15 029
**Average**	**0.86**	0.03	0.09	0.06	0.03	**0.83**	0.02	0.13	0.06	0.04	**0.81**	0.02	0.15	0.06	0.05	15 159
**Profiles**	**HER2ST: 500 Genes**															**NoG**
	**70% Downsampled**					**50% Downsampled**					**30% Downsampled**					
	**M1**	**M2**	**M3**	**M4**	**M5**	**M1**	**M2**	**M3**	**M4**	**M5**	**M1**	**M2**	**M3**	**M4**	**M5**	
A1	**0.87**	0.02	0.11	0.11	0.07	**0.77**	0.01	0.15	0.12	0.09	**0.81**	0.01	0.17	0.12	0.09	15 045
E1	**0.87**	0.05	0.20	0.10	0.08	**0.77**	0.04	0.23	0.10	0.08	**0.81**	0.04	0.27	0.11	0.09	15 701
F1	**0.80**	0.03	0.08	0.03	0.02	**0.84**	0.03	0.12	0.03	0.03	**0.83**	0.02	0.14	0.04	0.03	14 861
H1	**0.86**	0.03	0.15	0.13	0.07	**0.81**	0.02	0.20	0.14	0.09	**0.80**	0.01	0.25	0.15	0.13	15 029
**Average**	**0.85**	0.03	0.14	0.09	0.06	**0.80**	0.03	0.17	0.10	0.07	**0.81**	0.02	0.21	0.10	0.09	15 159
**Profiles**	**spatialLIBD: 10000 Genes**															**NoG**
	**70% Downsampled**					**50% Downsampled**					**30% Downsampled**					
	**M1**	**M2**	**M3**	**M4**	**M5**	**M1**	**M2**	**M3**	**M4**	**M5**	**M1**	**M2**	**M3**	**M4**	**M5**	
151507	**0.75**	0.00	0.15	0.04	0.05	**0.82**	0.00	0.17	0.04	0.05	**0.53**	0.00	0.19	0.04	0.06	33 538
151510	**0.84**	0.00	0.14	0.04	0.05	**0.81**	0.00	0.17	0.04	0.05	**0.86**	0.00	0.18	0.04	0.06	33 538
151669	**0.35**	0.03	0.14	0.06	0.06	**0.73**	0.03	0.16	0.06	0.07	**0.22**	0.03	0.13	0.07	0.07	33 538
151673	**0.90**	0.02	0.19	0.08	0.07	0.16	0.02	**0.22**	0.08	0.08	**0.80**	0.02	0.23	0.08	0.09	33 538
**Average**	**0.70**	0.01	0.15	0.06	0.06	**0.56**	0.01	0.18	0.06	0.06	**0.59**	0.01	0.18	0.06	0.07	33 538
151507	**0.64**	0.01	0.32	0.09	0.08	**0.67**	0.00	0.35	0.09	0.08	**0.68**	0.01	0.36	0.10	0.09	33 538
151510	**0.65**	0.01	0.32	0.11	0.09	**0.65**	0.01	0.35	0.11	0.09	**0.66**	0.01	0.36	0.12	0.10	33 538
151669	**0.74**	0.01	0.29	0.13	0.09	**0.77**	0.01	0.32	0.12	0.10	**0.77**	0.01	0.24	0.13	0.11	33 538
151673	**0.74**	0.03	0.46	0.22	0.18	**0.70**	0.02	0.48	0.22	0.20	**0.73**	0.02	0.49	0.23	0.21	33 538
**Average**	**0.69**	0.01	0.35	0.14	0.11	**0.70**	0.01	0.37	0.14	0.12	**0.71**	0.01	0.36	0.14	0.13	33 538

Bold values indicate the highest PC value among the compared methods for each dataset, gene set, and downsampling condition.

#### Ablation study

To assess the impact of multimodal regularization, we compared a reduced version of STORM using only the magnitude regularization term (R1) with the full model that integrates all proposed regularization components (R1–R4). As shown in Table 2, relying solely on the R1 term is insufficient to capture the underlying structure of spatial gene expression. The full regularization model (R1–R4) consistently improves reconstruction performance across different samples, number of genes and downsampling rates. We observe that the contribution of the regularization terms to the accuracy of STORM is higher for larger sampling rates. This demonstrates that STORM’s integration strategy of multimodal biological data is essential especially when less observations are available.

**Table 2:** Quantitative comparison of reconstruction performance (Pearson correlation) between the R1-only setting and the full regularization model (R1–R4) across different downsampling rates (30%, 50%, and 70%) for the top 500 and top 10 000 highly variable. Our results highlight the impact of incorporating additional regularization terms

**500 genes**					
**Sample**	**Regularization Setting**	**Downsampling rate**			**Avg**
		**30%**	**50%**	**70%**	
**E1**	R1 only	0.7704	0.6808	0.7355	**0.7289**
	All regularizers	0.8064	0.7734	0.8716	**0.8171**
**151 510**	R1 only	0.5860	0.5434	0.4936	**0.5410**
	All regularizers	0.6600	0.6519	0.6458	**0.6526**
**TENX72**	R1 only	0.2578	0.2564	0.1449	**0.2196**
	All regularizers	0.2830	0.3190	0.2610	**0.2877**
**MEND90**	R1 only	0.4377	0.3986	0.3248	**0.3870**
	All regularizers	0.5311	0.5182	0.5004	**0.5166**
**10 000 genes**					
**Sample**	**Regularization Setting**	**Downsampling rate**			**Avg**
		**30%**	**50%**	**70%**	
**E1**	R1 only	0.7705	0.6808	0.7355	**0.7289**
	All regularizers	0.7716	0.8456	0.8571	**0.8248**
**151 510**	R1 only	0.8171	0.7910	0.7640	**0.7907**
	All regularizers	0.8364	0.8110	0.8585	**0.8353**
**TENX72**	R1 only	0.4362	0.4116	0.3179	**0.3886**
	All regularizers	0.4991	0.5060	0.5017	**0.5023**
**MEND90**	R1 only	0.4266	0.3822	0.2993	**0.3694**
	All regularizers	0.5674	0.5516	0.5436	**0.5542**

Boldface shows the average PC value across all downsampling rates when only R1 regularizer is used and that when all the regularizers are used.

#### Computational efficiency and scalability analysis

To assess the practical applicability of STORM to large-scale ST analysis, we evaluated its computational efficiency in terms of running time (s) and peak memory (RAM). We compared STORM with representative competing methods, including STAGE, iStar, Nicheformer, and Novae, under two gene-set settings (500 and 10 000 genes). All methods were evaluated under a unified experimental setup, and the resulting running time and memory comparisons are summarized in Table 3.

**Table 3:** Running time and memory usage averaged over HEST-1k, HER2ST, and spatialLIBD datasets of five methods; STORM, iStar, STAGE, Nicheformer, and Novae for 500 and 10,000 genes

**500 Genes**		
**Method**	**Running time (s)**	**Peak memory (GB)**
STORM	57.30	8.53
iStar	1455.69	38.70
STAGE	26.10	3.12
Nicheformer	3.83	2.79
Novae	46.75	2.50
**10 000 Genes**		
**Method**	**Running time (s)**	**Peak memory (GB)**
STORM	82.02	8.90
iStar	1737.62	38.70
STAGE	59.60	3.94
Nicheformer	10.24	2.80
Novae	48.10	2.58

Compared with STAGE, STORM requires higher running time and memory. This is expected because STORM incorporates tissue morphology and gene–gene interaction structure, whereas STAGE is coordinate-driven. STORM nevertheless remains substantially more efficient than iStar, whose HIPT-based whole-slide feature extraction results in much higher memory usage.

#### Evaluation of spot-level accuracy and spatial consistency

**Figure 4:**
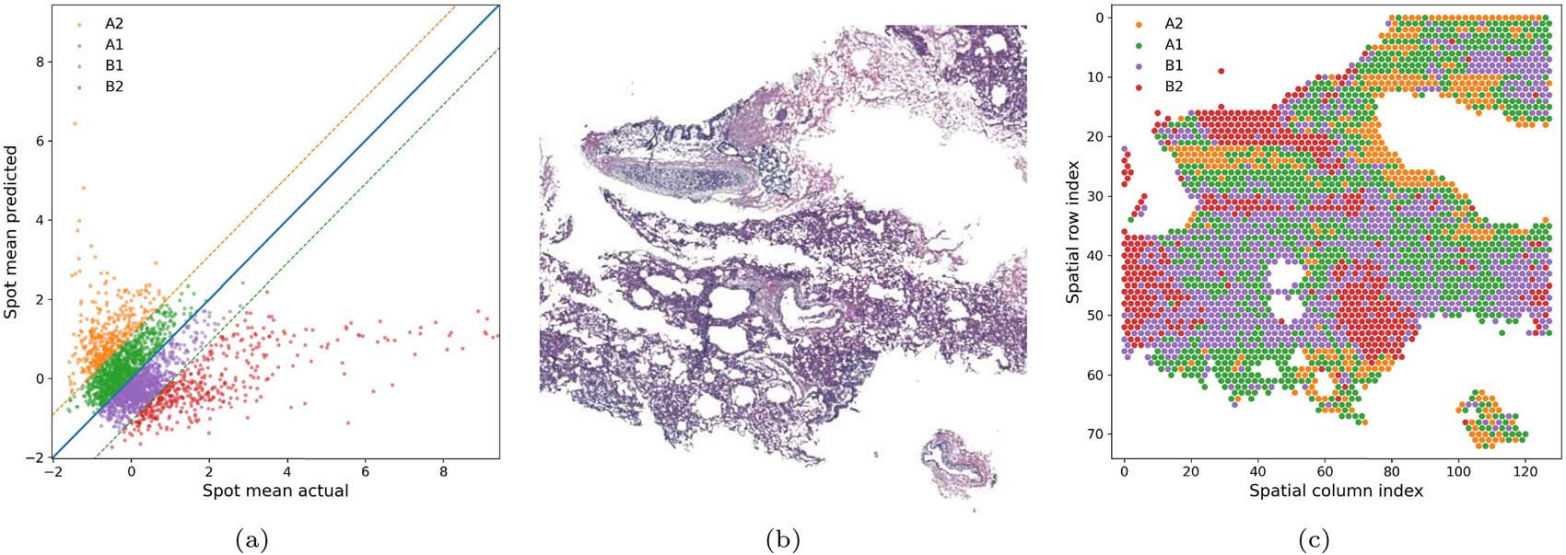
Biologically structured spatial distribution of reconstruction uncertainty. (a) Mean predicted expression per spatial spot versus mean ground-truth expression per spot (log1p-transformed and robustly scaled) for the MEND90 lung tissue profile, with each point representing an individual spatial location. The central diagonal indicates perfect agreement between predicted and true transcriptional values, while the two flanking diagonals denote deviations of *± 1.0*. These thresholds partition the spatial spots into four reconstruction regimes: A1 (accurate or mildly overestimated), A2 (strongly overestimated), B1 (mildly underestimated), and B2 (strongly underestimated). (b) Corresponding whole-slide histology image, providing anatomical and morphological context for spatial interpretation of prediction errors. (c) Spatial mapping of the four error regimes across the tissue section reveals a non-uniform distribution of reconstruction uncertainty. Higher-magnitude errors are preferentially localized to tissue boundaries and regions of elevated transcriptional activity, which are known to exhibit increased cellular diversity, dynamic cell–cell interactions, and complex microenvironmental structure. In contrast, regions of homogeneous tissue architecture show predominantly low-error predictions. Together, these observations indicate that reconstruction uncertainty in STORM is not randomly distributed but instead aligns with biologically complex tissue regions, suggesting that the model preserves coherent spatial transcriptional patterns while appropriately reflecting intrinsic biological variability and heterogeneity inherent to spatially resolved transcriptomic data.

Thus far, we have evaluated prediction accuracy by assessing the correlation between predicted and true transcription values aggregated across genes. We now analyze prediction errors at the level of individual spatial locations to better understand how the reconstruction quality varies across tissue regions. Given an ST profile with a subset of masked spots, we first used STORM to estimate the transcription values at hidden locations. We then compared the predicted and ground-truth expression values across all gene–spot combinations. Figure 4 (left) illustrates this distribution for the MEND90 profile at a 30% masking rate. To facilitate interpretation, we categorized the spots into four groups labeled A1, A2, B1, and B2. Categories A and B correspond to the overestimated and underestimated spots, respectively, whereas indices 1 and 2 denote low- and high-error predictions, respectively. Accurately predicted spots were included in category A1 (low-error overestimation) without loss of generality. Overall, STORM achieved high reconstruction accuracy for the majority of spatial locations, with *∼ *70.0% of spots falling within an error interval of *± 1*. Among these, 50.0% of the spots were slightly overestimated, with 36.6% exhibiting low error, while 33.4% were underestimated with similarly low error. High-error predictions occur less frequently and are primarily associated with spots that exhibit very high transcriptional values. Figure 4 (right) shows the spatial distribution of the prediction errors with respect to the *x*- and *y*-coordinates of the tissue. These results suggest that higher reconstruction errors tend to occur near tissue boundaries and at locations with elevated transcriptional activity. One possible explanation is the increased cellular heterogeneity at the tissue boundaries, which may be more challenging to accurately model. Alternatively, boundary regions may be subjected to higher measurement noise in spatial transcriptomic assays. In addition, boundary spots benefit from fewer neighboring locations, which may limit the information available to the WSI alignment regularizer ($R_{2}$) and the spatial smoothness term ($R_{3}$) compared with the interior tissue regions.

#### Evaluation of the spatial transcriptomics recovery

To qualitatively assess the ability of STORM to recover biologically meaningful spatial transcriptomic signals, we examined the reconstructed expression maps for representative genes under increasing levels of spot-level sparsity. Figure 6 illustrates this analysis using BPIFB1 in the MEND90 lung tissue profile is known to exhibit spatially structured expression patterns associated with airway epithelial regions. When a substantial fraction of spatial locations was masked, the observed expression maps become highly fragmented and failed capture the underlying spatial organization of transcriptional activity (Fig. 6a, left). Particularly, increasing levels of missingness progressively disrupt contiguous expression domains, obscuring biologically relevant gradients and regional patterns. This sparsity-induced degradation highlights the intrinsic difficulty of ST recovery and underscores the need for models that can leverage both spatial and biological contexts. Following reconstruction with STORM, the recovered expression maps exhibited markedly improved spatial coherence across all masking levels (Fig. 6a, right panel). Notably, STORM restores continuous expression domains that closely resemble the ground-truth spatial distribution, even when *∼ *70% of the spatial spots are unobserved. Rather than producing overly smooth or uniform estimates, the reconstructed maps preserve sharp spatial transitions and localized high-expression regions, indicating that the model captures a fine-grained spatial structure rather than merely interpolating the missing values. Zoomed-in analyses of spatially complex regions further demonstrated the fidelity of reconstruction (Fig. 6c). In these regions, which are characterized by rapid local changes in expression intensity, STORM accurately recovers heterogeneous transcriptional patterns that are lost in sparsely observed data. The recovered profiles aligned closely with the ground-truth expression both in terms of spatial extent and relative intensity, suggesting that the model successfully integrated local spatial smoothness with gene-specific variability. Importantly, the recovered expression patterns remained biologically plausible across the entire tissue section. High-expression regions are preserved in anatomically consistent locations, whereas boundary regions are known to exhibit increased cellular heterogeneity and measurement noise—display-controlled uncertainty rather than spurious signal amplification. This behavior reflects the combined effect of STORM’s multimodal regularization, in which spatial smoothness, histological context, and gene–gene interaction structure jointly constrain the reconstruction. Together, these results demonstrate that STORM performs true spatial transcriptomic recovery rather than simple smoothing or nearest-neighbor interpolation. By reconstructing missing expression values in a manner that respects tissue architecture and biological organization, STORM enables reliable downstream spatial analyses even under severe data sparsity, substantially extending the practical utility of ST datasets.

#### Effect of whole-slide image homogeneity on spatial transcriptomics recovery

Next, we evaluated the impact of spatial homogeneity in whole-slide image on the accuracy of gene–expression prediction in spatial transcriptomic profiles. The objective of this analysis was to assess whether variability in WSI-derived spatial features contributes to distinguishing between localized transcriptional variations. We quantified spatial homogeneity using Moran’s *I* [22] and Geary’s *C* [23], which are two widely used spatial autocorrelation metrics. Moran’s *I* captures global spatial smoothness, with higher values indicating stronger global autocorrelation, whereas Geary’s *C* measures local similarity, with lower values corresponding to higher local homogeneity. After min–max normalization across samples, the two metrics are directionally aligned such that higher values consistently indicate greater spatial homogeneity. Specifically, normalized Moran’s *I* is used directly, whereas normalized Geary’s *C* is inverted. The resulting spatial homogeneity score, denoted by $H_{2}$, is computed as 


**Figure 5:**
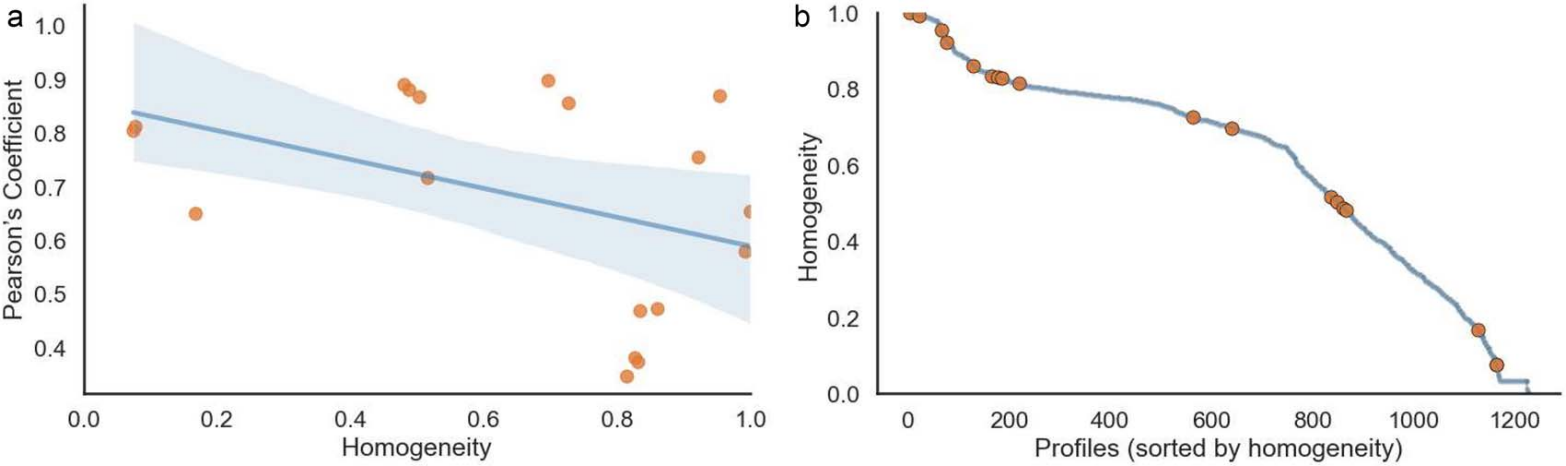
Histological spatial heterogeneity modulates reconstruction performance. (a) Association between STORM reconstruction accuracy and histological spatial homogeneity across all evaluated HEST-1k ST profiles. Reconstruction accuracy is measured by PC between reconstructed and ground-truth gene–expression values. The solid line represents the linear regression fit, with the shaded region indicating the 95% confidence interval. Higher homogeneity scores correspond to globally smoother whole-slide images (WSIs), whereas lower scores reflect increased local morphological variability. The negative association suggests that STORM benefits from spatially heterogeneous histological features, which provide informative cues for resolving localized transcriptional variation. (b) Distribution of spatial homogeneity scores across all profiles in the HEST-1k dataset, sorted in descending order. HEST-1k profiles included in this study are highlighted with orange circles, illustrating that the evaluation set spans a wide range of histological complexity and is not restricted to highly heterogeneous tissues.

\begin{align*} & H_{2} = \frac{I + (1 - C)}{2}, \end{align*}

where *I* and *C* represent normalized Moran’s *I* and Geary’s *C* values, respectively. Higher $H_{2}$ values correspond to more spatially homogeneous WSIs. We hypothesized that STORM achieves higher prediction accuracy for profiles with lower $H_{2}$ values, as increased spatial variability in WSI features provides richer morphological cues for reconstruction. Figure 5 (left) illustrates the relationship between WSI homogeneity and PC coefficient of the predicted gene–expression values across all evaluated profiles. These results support our hypothesis, showing a general trend of higher PC values for profiles with lower $H_{2}$. However, a wide range of PC values was observed even among highly homogeneous profiles, indicating that the absence of strong local visual variability does not necessarily limit the performance of STORM. This observation suggests that STORM can compensate for reduced WSI variability by leveraging complementary information from other modalities, particularly gene–gene interaction structures. Figure 5 (right) shows the distribution of $H_{2}$ values across all 1229 profiles in the HEST-1k dataset. The distribution reveals that a large majority of the profiles (*>73%*) exhibit $H_{2}$ values <0.8, indicating substantial spatial heterogeneity in their WSIs. Consequently, STORM is expected to provide high-quality ST reconstruction for most profiles in the dataset. Importantly, the subset of profiles used in our evaluation is approximately evenly distributed between $H_{2} \leq 0.8$ and $H_{2}> 0.8$, demonstrating that the strong performance of STORM is not limited to profiles with highly variable WSI features, but generalizes across a broad range of tissue homogeneity levels.

**Figure 6:**
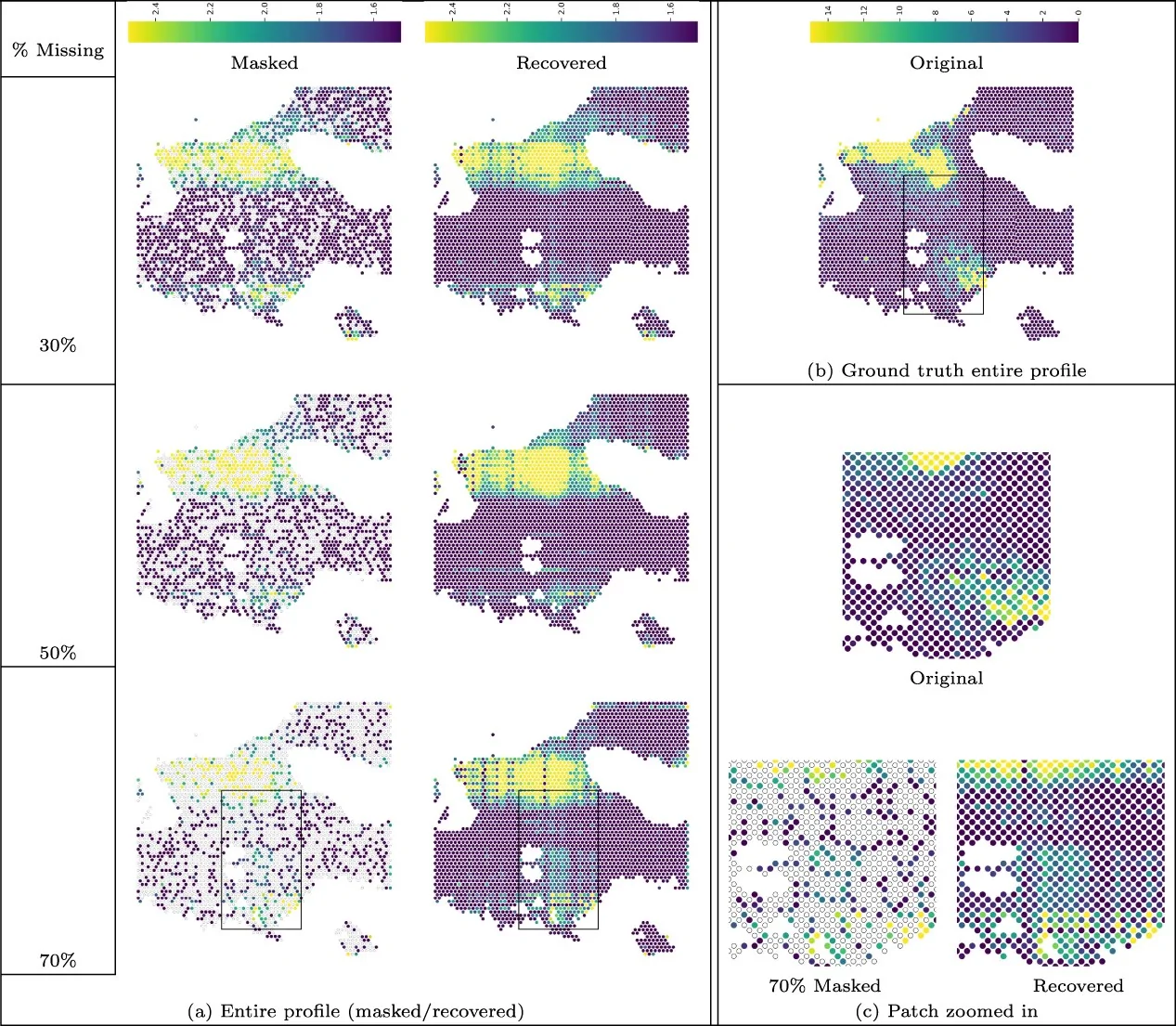
Reconstruction of localized, high-variability spatial expression patterns, with heatmaps illustrating the spatial expression of the BPIFB1 gene in the MEND90 lung tissue profile under increasing levels of data sparsity: (a) observed expression maps after random masking of spatial spots, together with the corresponding STORM-reconstructed expression maps, with unobserved spots shown in light gray; (b) ground-truth spatial expression map for BPIFB1 in the same tissue section, with highlighted bounding boxes in (a) and (b) indicating spatially confined regions exhibiting complex and highly heterogeneous expression patterns; (c) zoomed-in views of one representative high-variability region, where the top panel shows the ground-truth expression, the bottom-left panel shows the sparsely observed expression map with 70% of spots masked, and the bottom-right panel shows the corresponding reconstruction produced by STORM.

## Conclusion

In this study, we introduced STORM, a biologically informed tensor decomposition framework for completing and enhancing spatial transcriptomic profiles under substantial missing-data conditions. By formulating spatial transcriptomic recovery as a low-rank tensor completion problem augmented with multimodal regularization, STORM explicitly integrates spatial proximity, tissue morphology derived from whole-slide images, and gene–gene interaction structure into a unified optimization objective. This design enables the robust reconstruction of spatial gene–expression patterns while preserving biological coherence across genes and tissue regions. Comprehensive experimental evaluations on diverse lung tissue profiles demonstrated that STORM consistently outperforms state-of-the-art methods across a wide range of masking ratios, gene set sizes, and tissue types. In particular, STORM exhibits strong robustness to extreme sparsity, maintaining high reconstruction accuracy even when *∼ *70% of the spatial locations are missing. Detailed spot-level and gene-level analyses further revealed that the model produces spatially coherent reconstructions, with errors concentrated primarily in biologically challenging regions such as tissue boundaries and high-expression outliers. Importantly, our analysis of WSI homogeneity shows that, while local visual variability enhances reconstruction accuracy, STORM remains effective even for spatially homogeneous tissues by leveraging complementary biological priors encoded in gene interaction networks. Beyond its empirical performance, STORM provides a principled framework for integrating heterogeneous data modalities within ST analysis. The modular structure of its regularization terms allows the flexible incorporation of additional biological knowledge sources, such as cell-type annotations, pathway-level constraints, or multi-omics measurements. While the current study focused on transcript-level reconstruction, the proposed framework naturally extends to higher-resolution settings and other spatially resolved molecular modalities. Taken together, STORM advances the state of ST reconstruction by bridging statistical tensor modeling with biologically grounded constraints. We anticipate that this approach will facilitate more accurate downstream analyses of tissue organization, cellular heterogeneity, and disease microenvironments, ultimately supporting improved biological interpretation and translational insights from spatially resolved genomic data.

**Key Points**

- Spatial transcriptomics optimization by resolution via matrix factorization (STORM) is a biologically informed tensor decomposition framework designed to reconstruct spatial transcriptomics (ST) data under conditions of severe sparsity and missing measurements.
- STORM jointly integrates spatial smoothness across neighboring locations with histological morphology from whole-slide images and gene–gene interaction structure within a unified optimization framework.
- By embedding multimodal biological priors directly into the reconstruction process, STORM preserves spatial coherence and functional gene relationships beyond approaches that rely only on image features or spatial coordinates.
- Extensive evaluations across multiple ST datasets, including HEST-1k, her2st, and spatialLIBD, demonstrate that STORM consistently outperforms state-of-the-art methods, maintaining high reconstruction accuracy even when *∼ *70% of spatial locations are missing.
- STORM provides a general and flexible foundation for high-resolution spatial transcriptomic analysis in settings where experimental measurements are incomplete or noisy.

## Supplementary Material

supplementary_materials_R1_bbag324

## Data Availability

This study uses the publicly available HEST-1k ST dataset [16] https://huggingface.co/datasets/MahmoodLab/hest, her2st dataset [17] https://github.com/almaan/her2st, and spatialLIBD dataset [18] https://research.libd.org/spatialLIBD/. No new experimental data were generated; missing spatial measurements were synthetically introduced and subsequently reconstructed using the proposed method. The source code supporting the findings of this study is publicly available at https://github.com/denizgurarslan/STORM.
