# Correction: STORM: spatial transcriptomics optimization by resolution via matrix factorization

**DOI:** 10.1093/bib/bbag604

**Published:** 2026-10-20

This is a correction to: Deniz Gurarslan, Oscar Camargo, Omer Zeyveli, Yasin Almalioglu, Yanjun Li, Mehmet Turan, Tamer Kahveci, STORM: spatial transcriptomics optimization by resolution via matrix factorization, *Briefings in Bioinformatics*, Volume 27, Issue 3, May 2026, bbag324, https://doi.org/10.1093/bib/bbag324

Material in the supplementary data file has now been corrected (in section ‘**Integration of protein-protein interaction topology.**’ The fifth sentence was removed: “The resulting interaction graph is inherently unsigned, since STRING scores represent confidence of functional associations aggregated from multiple evidence sources rather than explicit activating or inhibitory relationships.”) and the supplementary data file resupplied to the article.

