# Supplementary material for: STORM: spatial transcriptomics optimization by resolution via matrix factorization
Source: Brief Bioinform. 2026 Jun 22;27(3):bbag324. doi: 10.1093/bib/bbag324 (PMC13284717; doi:10.1093/bib/bbag324)
Supplement: supplementary_materials_R1_bbag324 [file supplementary_materials_r1_bbag324.pdf]

Supplementary materials

SM1. Details of the STORM algorithm

Notation and Problem Definition

Let I and J denote the number of spatial locations along the two spatial axes of a tissue section, and let K denote the number of genes profiled in a given spatial transcriptomics experiment. We define the index sets $Z_I = \{1, 2, \ldots, I\}$, $Z_J = \{1, 2, \ldots, J\}$, and $Z_K = \{1, 2, \ldots, K\}$. For each spatial location (i, j) with $i \in Z_I$ and $j \in Z_J$, and for each gene $k \in Z_K$, we denote the (unknown) transcriptional abundance of gene k at that location by $x_{i,j,k}$. Collectively, the spatial transcriptomics profile can be viewed as a three-dimensional array indexed over space and genes. We define the complete index set of all possible spatial–gene combinations as

$$\Psi = Z_I \times Z_J \times Z_K.$$

In practice, because of intrinsic limitations of ST technologies—including dropouts, limited capture efficiency, and variable tissue quality—expression measurements are available only for a subset of these indices. We denote by $\Omega \subseteq \Psi$ the set of observed triplets for which transcriptional values are measured. The complement set $\Psi \setminus \Omega$ corresponds to unobserved or missing gene expression values- that may arise from both technical sparsity and biological heterogeneity. The objective compltein of spatial transcriptomics completion is to infer the missing entries $\{x_{i,j,k} : (i, j, k) \in \Psi \setminus \Omega\}$ given the observed measurements indexed by Ω. Importantly, this problem is not merely a numerical interpolation: the goal is to recover transcriptional values in a manner that preserves biologically meaningful spatial structure, respects tissue morphology, and maintains coherent gene–gene relationships across the reconstructed spatial domain. The STORM framework addresses this challenge by leveraging shared low-dimensional structure across spatial locations and genes, together with biologically informed constraints that guide the reconstruction of plausible and interpretable solutions.

Tensor Decomposition Framework

We formulate the spatial transcriptomics (ST) completion problem as a tensor decomposition task that explicitly captures the multidimensional structure of spatial gene expression data. Using the notation introduced above, we represent an ST profile as a third-order tensor

$$X \in \mathbb{R}^{I \times J \times K},$$

where the first two modes correspond to the two-dimensional spatial organization of the tissue and the third mode corresponds to gene identity. Each tensor entry x_{ijk} represents the transcriptional abundance of gene k at spatial location (i, j). In practice, owing to technical sparsity and measurement noise, only a subset of tensor entries indexed by $(i, j, k) \in \Omega$ is observed, whereas the remaining entries are missing. Modeling spatial transcriptomics data as a tensor provides a natural representation that preserves intrinsic coupling between spatial coordinates and gene expression. Unlike matrix-based formulations that collapse spatial dimensions or treat genes independently, tensor formulation enables joint modeling of spatial structures along both axes together with gene-specific variation. This is particularly important in spatial transcriptomics, where transcriptional programs are shaped by a coordinated spatial organization and shared molecular pathways. To exploit this structure, we adopted a low-rank tensor decomposition framework based on the canonical polyadic (CP) model. Specifically, we approximate the tensor X using three factor matrices:

$$A \in \mathbb{R}^{I \times R}, \quad B \in \mathbb{R}^{J \times R}, \quad C \in \mathbb{R}^{K \times R},$$

where R denotes the tensor rank and reflects the dimensionality of the latent representation. Matrices A and B encode latent spatial factors along the two spatial axes, whereas matrix C captures latent gene-specific patterns shared across the tissue. Under CP decomposition, the tensor is approximated as

$$X \approx A \circ B \circ C,$$

where $\circ$ denotes the outer product. Accordingly, each transcriptional measurement is modeled as

$$\hat{x}_{ijk} = \sum_{r=1}^{R} a_{ir}\, b_{jr}\, c_{kr},$$

which represents the interaction between spatial latent factors and gene-specific latent components. This formulation naturally couples gene expression across spatial locations and genes through a shared low-dimensional structure, enabling information to propagate from the observed entries to unobserved regions. In classical tensor completion, the factor matrices are estimated by minimizing the reconstruction error over the observed entries.

$$\mathcal{L}_{\text{recon}} = \sum_{(i,j,k) \in \Omega} \left(x_{ijk} - \hat{x}_{ijk}\right)^2.$$

While this objective encourages the accurate fitting of observed data, it does not, by itself, enforce biological plausibility or spatial coherence in the recovered tensor. In the context of spatial transcriptomics, purely statistical low-rank reconstruction can yield solutions that fit the data numerically but fail to respect tissue architecture, spatial continuity, or functional gene relationships. STORM extends this classical formulation by augmenting the reconstruction objective with biologically informed regularization terms that explicitly encode spatial smoothness, tissue morphology, and gene–gene interaction structure. These components guide low-rank decomposition toward biologically coherent solutions, enabling the robust recovery of missing transcriptional values while preserving the spatial and molecular organization of the tissue.

STORM tensor decomposition model

The STORM framework is built upon a set of explicit modeling assumptions that reflect the fundamental biological and technical properties of spatial transcriptomic data. These assumptions guide the construction of the optimization objective and determine how complementary spatial, morphological, and molecular information is incorporated into the tensor decomposition process. Rather than relying on purely statistical reconstruction, STORM embeds biologically motivated constraints directly into the loss function, ensuring that recovered expression profiles remain consistent with the known principles of tissue organization and gene regulation. At a high level, the STORM objective comprises a weighted reconstruction term coupled with multiple regularization components, each encoding a distinct aspect of spatial transcriptomic structure. The reconstruction term accounts for heterogeneity in gene expression reliability across spatial locations, whereas regularizers impose constraints that promote morphological consistency with histology images, spatial smoothness across neighboring tissue regions, and functional coherence among interacting genes. Together, these elements enable the model to balance data fidelity with biological plausibility. In the following sections, we introduce the first modeling assumption underlying the STORM objective, which motivates the design of the weighted reconstruction loss. The remaining assumptions were subsequently formalized through the definition of dedicated regularization terms, each corresponding to a specific modality or biological prior integrated into the framework.

Overview. The first modeling assumption underlying STORM acknowledges a fundamental property of spatial transcriptomics data: genes differ substantially in both their expression magnitude and the degree to which their transcriptional activity is spatially structured across tissue regions. Although many genes exhibit low expression levels or relatively uniform spatial distributions, others display pronounced spatial heterogeneity that reflects localized biological processes, cellular niches, or microenvironmental gradients. Treating all genes as equally informative in the reconstruction objective can therefore bias the optimization of weakly informative or spatially uniform signals, thereby diluting biologically meaningful spatial patterns. To account for this heterogeneity, STORM formulates the reconstruction objective as a gene-weighted mean squared error (WMSE) evaluated over in-tissue spatial locations. This design prioritizes genes that encode strong and spatially variable transcriptional signals, which are more informative for recovering the spatial structure, while reducing the influence of genes whose expression provides limited spatial discrimination. Importantly, this weighting strategy does not discard low-variance genes; rather, it modulates their contribution to loss in proportion to their spatial informativeness. Formally, for each gene $k \in Z_K$, let Ω_k denotes the set of spatial locations (i, j) at which the transcriptional value of gene k is observed. Using these locations, we first computed the mean expression level of gene k as follows:

$$\mu_k = \frac{1}{|\Omega_k|} \sum_{(i,j)\in\Omega_k} x_{ijk}.$$

Next, we quantified the degree to which each gene exhibited a spatially structured transcriptional variation across tissues. For a given gene k, we compute its spatial variance over the observed in-tissue locations as

$$\sigma_k^2 = \frac{1}{|\Omega_k|} \sum_{(i,j)\in\Omega_k} (x_{ijk} - \mu_k)^2 .$$

This quantity captures the extent to which the expression of gene k varies across space and serves as a proxy for its potential to encode biologically meaningful spatial patterns. However, directly using raw variance values for weighting can, lead to numerical instability and overemphasis of a small number of extremely variable genes. To mitigate this effect while preserving relative differences in spatial variability, we applied logarithmic transformation followed by normalization across genes. Specifically, we define gene-specific weight w_k as

$$w_k = \frac{\log(1 + \sigma_k^2)}{\max_{k\in Z_K} \log(1 + \sigma_k^2)}.$$

Logarithmic scaling compresses the dynamic range of variances, preventing highly variable genes from disproportionately dominating the optimization, while normalization ensures that all weights lie within a comparable range.

Using these gene-specific weights, we define the weighted mean squared error (WMSE) reconstruction loss as

$$\mathcal{L}_{\text{WMSE}} = \frac{\sum_{(i,j,k)\in\Omega} w_k\,(x_{ijk} - \hat{x}_{ijk})^2}{\sum_{(i,j,k)\in\Omega} w_k}.$$

This formulation prioritizes genes whose expression exhibits pronounced and spatially structured variability, which is more informative for reconstructing spatial organization, while still retaining contributions from genes with lower variance. By modulating the influence of each gene according to its spatial informativeness rather than its absolute expression level, the reconstruction objective avoids being dominated by weakly informative or spatially uniform signals.

Consequently, the optimization process focuses on recovering biologically meaningful spatial structure and demonstrates increased robustness under varying levels of sparsity and dropout across tissues. Importantly, this weighting strategy enhances sensitivity to localized transcriptional programs without imposing hard thresholds or excluding genes from reconstruction, thereby maintaining transcriptome-wide coherence. STORM constructs a unified tensor decomposition framework by embedding key biological and structural properties of spatial transcriptomics data directly into the optimization objective through four complementary regularization terms, denoted by R_1, R_2, R_3, and R_4. Each regularizer encodes a distinct aspect of the biological organization that is not captured by low-rank reconstruction alone, enabling the model to move beyond purely statistical completion toward biologically grounded inference. Specifically, R_1 constrains the magnitude of the latent factor matrices to prevent overfitting

and to ensure stable low-rank representations. R_2 enforces the alignment between learned spatial latent representations and tissue morphology by integrating visual features extracted from the WSI. R_3 promotes spatial smoothness by encouraging coherent transcriptional transitions across neighboring tissue regions, reflecting the continuity of biological processes in space. Finally, R_4 incorporates prior biological knowledge by constraining gene representations according to known gene–gene interaction structure, thereby preserving functional coherence among reconstructed transcriptional programs. Together, these regularization components enable STORM to balance the reconstruction accuracy with biological interpretability across multiple spatial and molecular scales.

The relative contribution of each regularization term is controlled by the non-negative hyperparameters λ_1, λ_2, λ_3, and λ_4, which determine the trade-off between data fidelity and biological constraint enforcement. Figure 2 provides a schematic overview of the STORM framework, illustrating how multimodal biological information is integrated into the tensor decomposition model, and how each regularizer corresponds to a specific data modality or biological prior.

Under this formulation, the overall optimization objective is defined as

$$\mathcal{L}_{\text{total}} = \mathcal{L}_{\text{WMSE}} + \sum_{t \in \{1,2,3,4\}} \lambda_t R_t,$$

where the weighted reconstruction loss $\mathcal{L}_{\text{WMSE}}$ ensures the accurate fitting of the observed transcriptional measurements, and the regularization terms guide the solution toward biologically plausible spatial and molecular structure.

Having established the complete objective function and its multimodal regularization design, we next describe each regularization term—R_1, R_2, R_3, and R_4—in detail, highlighting the biological motivation and mathematical formulation underlying each component.

Overfitting avoidance. The regularization term R_1 is introduced to mitigate overfitting by penalizing large-magnitude entries in the latent factor matrices A, B, and C. Without such a constraint, the tensor decomposition may fit the observed entries with arbitrarily large parameter values, resulting in poor generalization to unobserved spatial locations. We define R_1 as

$$R_1 = \sum_{i,r} a_{ir}^2 + \sum_{j,r} b_{jr}^2 + \sum_{k,r} c_{kr}^2.$$

This form of ℓ_2 regularization is commonly used in classical tensor decomposition models and serves to control model complexity by discouraging excessively large latent representations, thereby improving stability and generalization.

Integration of WSI. To ensure that the learned spatial representations are consistent with the visual structure of the tissue, STORM aligns the spatial latent factors with features extracted from the corresponding whole-slide image. Consider the spatial location indexed by the ith row and jth column of an ST grid. Let $a_i^\top$ and $b_j^\top$ denote the latent vectors associated with the ith row of matrix A and the jth row of matrix B, respectively. We define the latent spatial embedding for this location as e_{ij}.

Spatial embedding is constructed via a rank-wise bilinear interaction of spatial factors,

$$e_{ij} = (a_{i1} b_{j1}, \ldots, a_{iR} b_{jR}),$$

which corresponds to the spatial component of CP decomposition. This embedding captures the latent spatial representation implied by tensor factors. To align this representation with tissue morphology, we projected WSI-derived visual features into the same latent space and enforced consistency through the regularization term R_2. Let N denote the total number of spatial locations at which the WSI information is evaluated, including both the observed and missing tissue regions. We collected spatial embeddings e_{ij} into a matrix

$$E_{\text{sp}} \in \mathbb{R}^{N \times R},$$

where each row corresponds to $e_{ij}^\top$ for a given spatial location. For each spatial location (i, j), we extract a 224×224 pixel patch centered at the corresponding tissue coordinates from the WSI and processed it using a ResNet50 network with the final fully connected layer removed. This produces a 2048-dimensional visual feature vector for each location. Stacking of these vectors yields a visual embedding matrix.

$$W \in \mathbb{R}^{N \times 2048},$$

ordered consistently with rows of E_{sp}.

To map the WSI-derived features into the latent spatial space, we learn the projection matrix

$$U \in \mathbb{R}^{2048 \times R}.$$

The projected visual representation is then given by $WU \in \mathbb{R}^{N \times R}$. The projection matrix U is learned jointly with the tensor factor matrices to align the projected visual features with spatial latent embeddings. The WSI alignment regularizer is defined as follows:

$$R_2 = \|E_{\text{sp}} - WU\|_F^2,$$

where $\|\cdot\|_F$ denotes the Frobenius norm. Minimizing R_2 encourages consistency between the latent spatial representations learned from the ST data and morphological patterns captured by the WSI.

$$R_2 = \|E_{\text{sp}} - WU\|_F^2. \tag{1}$$

Ensuring smoothness of spatial transitions. This regularization term formalizes the assumption that spatial proximity between tissue regions is associated with transcriptional similarity. Specifically, it penalizes abrupt transcriptional changes

between neighboring ST spots, thereby promoting spatial continuity in the reconstructed expression profiles. To model the spatial relationships among tissue locations, we constructed a graph-based Laplacian that captured the local geometric structure. We first built a k-nearest neighbor (kNN) graph using Euclidean distance on the two-dimensional spatial coordinates of all in-tissue locations, including both observed ST spots and missing regions ($k = 10$ in all experiments). Let $S \in \mathbb{R}^{N \times N}$ denote the adjacency matrix of this graph, where $S_{ij} = 1$ if spatial location i is connected to location j, and $S_{ij} = 0$ otherwise. Let D be the corresponding diagonal degree matrix with the entries $D_{ii} = \sum_j S_{ij}$. Using these definitions, we construct the normalized graph Laplacian as follows:

$$L_s = D^{-1/2}(D - S)D^{-1/2}. \tag{2}$$

This normalization balances the influence of spatial smoothing across locations with varying local connectivity and ensures stable propagation of information across tissues.

Let $E_{\text{full}} \in \mathbb{R}^{N \times R}$ denote the spatial latent embedding matrix over the entire tissue domain, where N includes all spatial locations (both observed and missing). The spatial smoothness regularizer is then defined using the trace operator $\text{Tr}(\cdot)$ as:

$$R_3 = \text{Tr}\left(E_{\text{full}}^{\top} L_s E_{\text{full}}\right). \tag{3}$$

Minimizing R_3 encourages spatially adjacent locations to have similar latent representations, thereby enforcing smooth transitions across the tissue domain and yielding spatially coherent and biologically meaningful reconstructed gene-expression maps.

Integration of protein–protein interaction topology. The final regularization term is motivated by the observation that the transcriptional activity of genes is not independent, but is shaped by functional interactions within gene regulatory and protein interaction networks. Genes that participate in shared biological pathways or molecular complexes tend to exhibit coordinated transcriptional behavior, reflecting their joint roles in cellular functions and biological processes. To capture this dependency structure, we modeled gene-gene relationships using a graph-based Laplacian constructed from a protein–protein interaction (PPI) topology. Interaction information was obtained from the STRING database [19], which provides experimentally validated and computationally inferred confidence scores for gene interactions. The resulting interaction graph is inherently unsigned, since STRING scores represent confidence of functional associations aggregated from multiple evidence sources rather than explicit activating or inhibitory relationships. To reduce the influence of noisy or weak associations, we retained only interactions with confidence scores greater than 0.65, ensuring that the resulting graph reflects high-confidence functional relationships. Let K_g denote the number of genes that retain at least one interaction after filtering. We constructed a weighted gene-gene adjacency matrix $G \in \mathbb{R}^{K_g \times K_g}$, where each entry $G(i, j)$ corresponds to the STRING confidence score associated with the interaction between the ith and jth genes. Let D_g be the corresponding diagonal degree matrix with entries $(D_g)_{ii} = \sum_j G(i, j)$. Using these definitions, we compute the normalized gene Laplacian as

$$L_g = D_g^{-1/2}(D_g - G)D_g^{-1/2}.$$

Based on this Laplacian, we define the gene interaction regularizer as

$$R_4 = \frac{1}{K_g} \text{Tr}\left(C^{\top} L_g C\right),$$

where $C \in \mathbb{R}^{K \times R}$ denotes the gene factor matrix of CP decomposition. To ensure biological relevance and dimensional consistency, R_4 is computed only over a subset of genes that are present in both the spatial transcriptomics tensor and STRING interaction network. Minimizing R_4 encourages genes that are functionally related within the PPI network to exhibit similar latent representations, thereby promoting biologically coherent structure in the reconstructed gene expression profiles and aligning the model output with the known patterns of gene co-regulation.

Adaptive regularization and loss scaling. The regularization coefficients λ_i control the relative contribution of the reconstruction loss and the biological regularizers R_i ($i \in \{1, \ldots, 4\}$). STORM initializes these coefficients with nominal values and subsequently refines them using an adaptive rescaling strategy to ensure that all the components of the objective function operate on comparable numerical scales. This normalization is critical for stable optimization, because the individual loss terms can differ substantially in magnitude. To specify the intended relative influence of each regularization component, STORM introduces user-defined proportionality constants P_i. The initial magnitudes of the reconstruction loss and regularization terms—computed using the initial values of the factor matrices A, B, and C—are used to determine the appropriate scaling factors for each λ_i. Through this adaptive adjustment, the optimization process is prevented from being dominated by any single term, enabling the balanced integration of reconstruction accuracy and biologically informed constraints.

To compute the adaptive regularization coefficients, we first evaluate the numerical scale of each loss component at initialization. Let WMSE denote the initial weighted mean squared reconstruction loss, and let R_i denote the initial value of the ith regularization term. These quantities serve as reference magnitudes for rescaling the objective function components. Each regularization coefficient λ_i is then computed by combining a user-specified proportionality constant P_i with the ratio between the reconstruction loss and the corresponding regularization term as follows:

$$\lambda_i = P_i \cdot \frac{\text{WMSE}}{R_i}, \qquad i \in \{1, 2, 3, 4\}.$$

This adaptive scaling strategy provides a principled mechanism for balancing the loss terms during optimization. By matching the scale of each regularization component to that of the reconstruction loss while preserving its relative importance, the method

prevents any single term from dominating the objective. Consequently, STORM achieves stable and well-conditioned optimization across datasets with varying levels of sparsity, spatial resolution, and gene dimensionality.

Choice of tensor rank. The tensor rank determines the number of latent components used in the decomposition and directly controls the expressive capacity of the model. Selecting an excessively high rank increases the risk of overfitting and incurs unnecessary computational costs, whereas an overly low rank constrains the model's representational power and leads to higher reconstruction error. Therefore, an appropriate rank requires balancing model flexibility with generalization. In STORM, we select the tensor rank to reflect the intrinsic dimensionality of the spatial transcriptomics data, guided by the number of genes included in the decomposition. This strategy enables the latent space to capture meaningful spatial and transcriptional structures without over-parameterizing the sparse ST datasets. In this study, we evaluated the model on two gene subsets: a reduced set consisting of the top 500 highly variable genes, and a larger set containing 10,000 genes. Based on empirical validation, we set the tensor rank to 15 for the 500-gene subset and 40 for the 10,000-gene subset. These values were found to provide a favorable trade-off between the reconstruction accuracy, robustness, and computational efficiency.

SM2. Theoretical foundations of STORM

In this Supplementary Material, we analyze the optimization behavior of the proposed STORM framework and establish convergence guarantees for the adopted training procedure. Owing to the joint presence of tensor factorization, a weighted reconstruction loss, and multiple structured regularization terms, the resulting optimization problem is inherently non-convex. Nevertheless, we demonstrate that the training procedure admits standard descent properties and converges to a stationary point under mild regularity assumptions.

For completeness and to ensure a self-contained presentation of the optimization analysis, we briefly recall the objective function introduced in the main manuscript. Let Θ denote the collection of all trainable parameters of the STORM model, including the CP factor matrices and associated projection parameters. Let $\hat{\mathcal{X}}(\Theta)$ denote the reconstructed tensor produced by the model, and let Ω denote the set of observed in-tissue spatial locations. For each gene k, let w_k denote the corresponding gene-specific weight as defined in the loss construction described in the methodology section of the main manuscript. Let $\mathcal{L}_{\mathrm{WMSE}}(\Theta)$ denote the weighted mean squared error (WMSE) reconstruction loss, and let $\mathcal{R}_i(\Theta)$, $i = 1, 2, 3, 4$, denote the structured regularization terms introduced in the main manuscript, each weighted by a non-negative regularization parameter λ_i.

With this notation in place, the STORM objective function is given by

$$\mathcal{L}(\Theta) = \mathcal{L}_{\mathrm{WMSE}}(\Theta) + \sum_{i=1}^{4} \lambda_i \mathcal{R}_i(\Theta). \tag{S1}$$

Here, X_{ijk} denotes the observed expression value of gene k at spatial location (i, j), and $\hat{X}_{ijk}(\Theta)$ denotes the corresponding reconstructed value produced by the STORM model parameterized by Θ. The index set Ω represents all observed in-tissue spatial locations used for training. The weighted mean squared error (WMSE) reconstruction loss is defined as

$$\mathcal{L}_{\mathrm{WMSE}}(\Theta) = \frac{\sum_{(i,j,k)\in\Omega} w_k \left(X_{ijk} - \hat{X}_{ijk}(\Theta)\right)^2}{\sum_{(i,j,k)\in\Omega} w_k}. \tag{S2}$$

This formulation assigns gene-specific importance to reconstruction errors while preserving scale invariance across genes. In the following, we analyze the optimization dynamics of the resulting objective function and establish convergence guarantees for the gradient-based training procedure adopted the STORM.

Property P1 (Lower boundary).

The objective function optimized by the STORM framework is lower bounded, which is a standard condition in non-convex optimization to rule out pathological cases where the objective decreases without bound and to ensure the existence of a minimizing sequence.

This condition is satisfied naturally in the STORM framework. The weighted reconstruction loss is formulated as a sum of squared residuals and is therefore non-negative by construction. In addition, all regularization terms correspond to non-negative quadratic forms: norm-based penalties are explicitly non-negative, whereas graph-based regularizers arise from normalized Laplacian matrices, that are positive semi-definite. Consequently, the overall objective function remains non-negative for all admissible parameter values.

Formally, this property can be expressed as

$$\inf_{\Theta} \mathcal{L}(\Theta) > -\infty. \tag{S3}$$

Property P2 (Smoothness on a Level Set).

We assumed that the objective function admits a Lipschitz-continuous gradient on a bounded level set.

Let $\Theta \in \mathbb{R}^p$ denote the vector collecting all trainable model parameters, and let $\mathcal{L}(\Theta)$ denote the corresponding objective function. Let Θ_0 denote the initialization of the optimization procedure, and define the associated level set as

$$\mathcal{S} = \{\Theta : \mathcal{L}(\Theta) \leq \mathcal{L}(\Theta_0)\}. \tag{S4}$$

Under appropriate step-size conditions, the objective value is non-increasing along the optimization trajectory; therefore, all iterations generated by the optimization algorithm remain within this level set.

We further assume that the gradient of the objective function is Lipschitz continuous on $\mathcal{S}$ with a Lipschitz constant $L > 0$. Here, $\nabla\mathcal{L}(\Theta)$ denotes the gradient of $\mathcal{L}$ with respect to all model parameters, evaluated at Θ, and let $\Phi \in \mathcal{S}$ denote another parameter vector in the same level set. Formally, this smoothness condition implies that for any $\Theta, \Phi \in \mathcal{S}$,

$$\|\nabla\mathcal{L}(\Theta) - \nabla\mathcal{L}(\Phi)\| \leq L\|\Theta - \Phi\|. \tag{S5}$$

This assumption is standard in non-convex optimization and is satisfied by the STORM objective. Each regularization term is a smooth quadratic function of the model parameters, including both norm-based penalties and graph Laplacian-induced quadratic forms. Moreover, the reconstruction loss corresponds to a weighted least-squares objective in the reconstructed tensor and is smooth with respect to the CP factors. Consequently, the gradient of the overall objective is Lipschitz continuous on the bounded level sets.

Gradient-Based Optimization.
We considered a gradient-based optimization scheme to minimize the STORM objective. Let $\Theta_t \in \mathbb{R}^p$ denote the vector collecting all trainable model parameters at iteration t, and let $\mathcal{L}(\Theta_t)$ denote the corresponding objective value. We assume a fixed step size $\eta > 0$.

Starting from an initial parameter vector Θ_0, the model parameters are iteratively updated according to the gradient descent rule

$$\Theta_{t+1} = \Theta_t - \eta\nabla\mathcal{L}(\Theta_t). \tag{S6}$$

Under the assumptions introduced above, this update scheme admits standard convergence guarantees, which we summarize as follows.

Proposition P3 (Monotone Descent and Convergence to Stationarity).
Assume that Properties P1–P2 hold and that the step size satisfies $0 < \eta \leq 1/L$. Subsequently, the sequence of iterates $\{\Theta_t\}$ generated by the gradient descent updates satisfies the following properties.

First, the objective value decreases monotonically along the optimization trajectory:

$$\mathcal{L}(\Theta_{t+1}) \leq \mathcal{L}(\Theta_t) - \frac{\eta}{2}\|\nabla\mathcal{L}(\Theta_t)\|^2. \tag{S7}$$

Moreover, the squared gradient norms are summable,

$$\sum_{t=0}^{\infty}\|\nabla\mathcal{L}(\Theta_t)\|^2 < \infty, \tag{S8}$$

this implies that the gradient norm converges to zero as the number of iterations increases.

$$\lim_{t\to\infty}\|\nabla\mathcal{L}(\Theta_t)\| = 0. \tag{S9}$$

Consequently, the sequence of iterations converges to the set of first-order stationary points of the STORM objective.

Proof of Proposition P3.
Under Property P2, the STORM objective admits an L-Lipschitz continuous gradient on the relevant level set and therefore satisfies the standard descent lemma for smooth non-convex functions. Choosing a step size $\eta \leq 1/L$ ensures a sufficient decrease of the objective at each iteration. Because the objective function is lower-bounded by Property P1, summing the descent inequality over iterations implies the summability of the squared gradient norms. Consequently, the gradient norm converges to zero, and the iterations converge to the set of first-order stationary points of the STORM objective.

SM3. Detailed Time and Space Complexity Analysis of STORM

We analyze the computational complexity of STORM by decomposing its reconstruction and regularization components. Let I and J denote the spatial grid dimensions, K the number of genes, R the factorization rank, D the histology feature dimension, N_{obs} the number of observed spots, N_{tissue} the number of in-tissue locations, and K_r the number of genes used in the gene regularizer.

Time Complexity.
The dominant cost arises from reconstructing the tensor $\hat{\mathcal{X}} \in \mathbb{R}^{I\times J\times K}$ via CP decomposition. Since each of the IJK entries aggregates R components, this step requires

$$O(IJKR).$$

The WSI-guided term consists of computing the spatial latent representation over the full grid,

$$O(IJR),$$

and aligning projected histology features with latent representations via WU, which costs

$$O(N_{\text{obs}}DR).$$

The spatial smoothness regularization relies on a dense Laplacian over in-tissue locations, resulting in

$$O(N_{\text{tissue}}^2 R),$$

while the gene interaction term uses a dense gene Laplacian with complexity

$$O(K_r^2 R).$$

Combining all components, the per-epoch complexity becomes

$$O\left(IJKR + IJR + N_{\text{obs}}DR + N_{\text{tissue}}^2 R + K_r^2 R\right),$$

and the total training cost over E epochs is

$$O\left(E \cdot \left(IJKR + IJR + N_{\text{obs}}DR + N_{\text{tissue}}^2 R + K_r^2 R\right)\right).$$

Space Complexity.
STORM stores the input, ground-truth, and reconstructed tensors, each requiring $O(IJK)$ memory. The factor matrices and projection matrix together require

$$O(IR + JR + KR + DR).$$

In addition, the spatial latent representation incurs a cost of

$$O(IJR),$$

and the observed histology features require

$$O(N_{\text{obs}}D).$$

The spatial and gene Laplacians introduce additional memory costs of

$$O(N_{\text{tissue}}^2) \quad \text{and} \quad O(K_r^2),$$

respectively.

Thus, the overall space complexity is

$$O\left(IJK + IJR + IR + JR + KR + DR + N_{\text{obs}}D + N_{\text{tissue}}^2 + K_r^2\right).$$

SM4. Robustness Analysis under Noise Perturbations

To assess the robustness of STORM to measurement noise, we introduce controlled perturbations to the observed gene expression values within the spatial transcriptomics tensor $X \in \mathbb{R}^{I \times J \times K}$.

We construct a perturbed tensor $\tilde{X}$ by injecting additive Gaussian noise in a gene-wise manner. Specifically, for each gene $k \in \{1, \dots, K\}$, we define a gene-specific scaling coefficient M_k as the maximum expression level of gene k, i.e.,

$$M_k = \max_{(i,j)} x_{i,j,k}.$$

The noise magnitude is controlled by a scaling factor $\beta \in \{0, 0.01, 0.02, 0.05, 0.1\}$.

The perturbed tensor entries are then given by

$$\tilde{x}_{i,j,k} = x_{i,j,k} + \beta \cdot M_k \cdot \epsilon_{i,j,k}, \quad \epsilon_{i,j,k} \sim \mathcal{N}(0, 1).$$

To preserve the intrinsic sparsity structure of spatial transcriptomics data, noise is added only to observed non-zero entries, i.e., for indices $(i, j, k) \in \Omega$ such that $x_{i,j,k} > 0$. Furthermore, to ensure biological plausibility, we enforce non-negativity of the perturbed values via element-wise clipping:

$$\tilde{x}_{i,j,k} = \max(0, \tilde{x}_{i,j,k}).$$

The STORM model is trained and evaluated using the noisy tensor $\tilde{X}$ without providing explicit information about the injected perturbations. Reconstruction accuracy is then measured with respect to the original (non-perturbed) tensor X, following the evaluation protocol described in Section 4.

We conduct experiments across multiple downsampling rates (30%, 50%, and 70%) and for two gene set sizes (500 and 10,000 highly variable genes), enabling a systematic assessment of robustness under varying levels of sparsity and noise.

As shown in Table 4, STORM demonstrates strong robustness to noise perturbations across different downsampling rates and samples. Reconstruction performance remains largely stable under low to moderate noise levels, particularly in the 500-gene setting. In the 10,000-gene setting, performance becomes more sensitive to increasing noise, as the inclusion of a larger number of genes introduces additional variability and amplifies the effect of perturbations; however, the model continues to preserve meaningful reconstruction accuracy even under higher noise levels.

Downsample Rate	MEND89 Noise percentage 500 genes 0%	1%	2%	5%	10%	10000 genes 0%	1%	2%	5%	10%
**70%**	0.4683	0.4722	0.4715	0.4684	0.4557	0.5026	0.4601	0.4598	0.3968	0.3855
**50%**	0.4575	0.4867	0.4866	0.4850	0.4763	0.5094	0.4919	0.4916	0.4186	0.4107
**30%**	0.4755	0.4937	0.4938	0.4926	0.4880	0.5147	0.5036	0.5035	0.4477	0.4410
**Average**	0.4671	0.4842	0.4840	0.4820	0.4733	0.5089	0.4852	0.4850	0.4210	0.4124

Downsample Rate	MEND90 Noise percentage 500 genes 0%	1%	2%	5%	10%	10000 genes 0%	1%	2%	5%	10%
**70%**	0.5433	0.5196	0.5214	0.5183	0.5072	0.5437	0.4601	0.4599	0.4582	0.4522
**50%**	0.5761	0.5656	0.5650	0.5606	0.5465	0.5516	0.4918	0.4916	0.4904	0.4858
**30%**	0.5805	0.5812	0.5808	0.5778	0.5655	0.5674	0.5036	0.5036	0.5027	0.4987
**Average**	0.5666	0.5555	0.5557	0.5522	0.5398	0.5542	0.4852	0.4850	0.4837	0.4789

Table 4. Reconstruction performance (Pearson correlation) under increasing noise levels (0%–10%) and different downsampling rates (30%, 50%, and 70%) for the MEND89 and MEND90 spatial transcriptomics samples. Results are reported for analyses based on 500 and 10,000 highly variable genes, demonstrating the robustness of the model under noisy observations.

Top 500 Highly Variable Genes

α	TENX72 70%	50%	30%	Avg	E1 70%	50%	30%	Avg	151510 70%	50%	30%	Avg
**0.01**	0.1545	0.2634	0.2628	0.2269	0.8764	0.7532	0.8038	0.8111	0.5158	0.5632	0.6008	0.5599
**0.1**	0.2012	0.2971	0.2895	0.2626	0.8716	0.7734	0.8064	**0.8171**	0.5802	0.6169	0.6388	0.6120
**1**	0.2610	0.3190	0.2830	**0.2877**	0.7398	0.5616	0.6526	0.6513	0.6459	0.6520	0.6601	**0.6526**
**10**	0.0396	0.0534	0.0323	0.0418	0.2600	0.2151	0.3690	0.2814	0.5376	0.5194	0.4589	0.5053
**100**	0.0010	0.0017	0.0019	0.0015	0.1120	0.0561	0.0980	0.0887	0.0683	0.0803	0.0822	0.0769

Top 10,000 Highly Variable Genes

α	TENX72 70%	50%	30%	Avg	E1 70%	50%	30%	Avg	151510 70%	50%	30%	Avg
**0.01**	0.3412	0.4250	0.4468	0.4043	0.8571	0.8456	0.7716	**0.8248**	0.7871	0.7990	0.8283	0.8048
**0.1**	0.4256	0.4721	0.4842	0.4606	0.9082	0.7030	0.6126	0.7413	0.8364	0.8110	0.8585	**0.8353**
**1**	0.5017	0.5060	0.4991	**0.5023**	0.8387	0.3908	0.3244	0.5180	0.7941	0.5398	0.7980	0.7106
**10**	0.0821	0.0461	0.0453	0.0579	0.4521	0.1384	0.1500	0.2468	0.3004	0.0381	0.2118	0.1834
**100**	0.0008	0.0004	0.0007	0.0006	0.1933	0.0826	0.0490	0.1083	0.0056	0.0053	0.0063	0.0057

Table 5. Sensitivity analysis of the global regularization scaling parameter α across three datasets for ST profiles TENX72 (HEST-1K dataset), E1 (HER2ST dataset), and 151510 (spatialLIBD dataset) and downsampling rates (30%, 50%, and 70%) using the top 500 and top 10,000 highly variable genes.

SM5. Sensitivity Analysis of the Scaling Parameter α

To provide practical guidance for applying the proposed scaling strategy, we further consider a global scaling parameter α that scales the overall contribution of the normalized regularization terms. These terms are adaptively normalized with respect to their initial magnitudes and the reconstruction loss. The adaptive regularization coefficients are defined as:

$$\lambda_i = P_i \cdot \frac{\text{WMSE}}{R_i}, \quad i \in \{1, 2, 3, 4\} \tag{4}$$

Building on this formulation, the final objective function incorporates α as:

$$\mathcal{L}_{\text{total}} = \mathcal{L}_{\text{WMSE}} + \alpha \sum_{i=1}^{4} \lambda_i R_i \tag{5}$$

To evaluate the effect of the global scaling parameter α on model performance, we conduct a systematic sensitivity analysis across a wide range of values. Specifically, we vary α from 0.01 to 100 and assess its impact under different downsampling ratios (30%, 50%, and 70%) across multiple spatial transcriptomics datasets.

The results of this analysis are summarized in Table 5. For the 500-gene setting, the best-performing α varies across datasets: $\alpha = 1$ achieves the highest performance for TENX72 and 151510, while $\alpha = 0.1$ performs best for E1. For the 10,000-gene setting, $\alpha = 1$ yields the best results for TENX72, whereas $\alpha = 0.01$ performs best for E1 and $\alpha = 0.1$ achieves the highest performance for

151510. These observations highlight that, while the optimal value of α may vary across datasets, strong performance is consistently achieved within a relatively small range of values.

Focusing on this range, $\alpha \in \{0.01, 0.1, 1\}$ produces stable and competitive results across all datasets. While the quantitative differences between these values are generally modest, $\alpha = 1$ often provides slightly more consistent performance across different settings, with $\alpha = 0.1$ serving as a strong alternative. Notably, $\alpha = 0.01$ also achieves comparable results in several cases, indicating stable performance under its original scaling. In contrast, larger values (e.g., $\alpha \geq 10$) consistently lead to performance degradation, suggesting over-regularization. We further encourage exploring nearby values to assess the effect of global scaling across different datasets

SM6. Limitations and Future Work

While STORM demonstrates strong performance and robustness across diverse spatial transcriptomics datasets, several limitations warrant discussion and point to promising directions for future research. First, STORM relies on a low-rank tensor decomposition framework that implicitly assumes that the underlying spatial–gene expression structure can be captured by a relatively compact latent representation. Although this assumption holds for the wide range of tissues and sparsity levels evaluated in this study, extremely heterogeneous tissues or highly localized transcriptional programs may require more expressive or adaptive latent models. Second, the integration of whole-slide images in STORM depends on the quality and resolution of the available histological data, as well as on the representational capacity of the pretrained visual encoder. In tissues with highly homogeneous morphology or in cases where the WSI quality is degraded, the contribution of image-based regularization may be limited. Future work could explore adaptive weighting or selective activation of visual regularization based on tissue-specific characteristics, as well as the use of foundation-scale vision models trained directly on histopathology data. Third, gene–gene interaction regularization relies on static protein–protein interaction networks derived from external databases. Although this prior provides a valuable biological context, such networks do not capture tissue-specific, condition-specific, or dynamic regulatory interactions. Incorporating context-aware interaction graphs, inferred directly from data or integrated from single-cell and multi-omics sources, represents an important direction for improving biological specificity. From a computational perspective, although STORM scales efficiently to large gene sets, the optimization of multiple coupled regularization terms introduces additional computational overhead compared with simpler interpolation-based methods. Future work will focus on accelerating optimization through stochastic or distributed training strategies, as well as exploring more efficient factorization schemes. Finally, while the present study focuses on reconstructing missing transcriptomic measurements, the STORM framework is not limited to this task. An important avenue for future research is the extension of STORM to joint modeling of multiple spatial modalities, such as spatial proteomics, epigenomics, or metabolomics, as well as to downstream tasks including cell-type deconvolution, spatial domain discovery, and disease-specific biomarker identification. Addressing these limitations will further enhance the flexibility and applicability of STORM, positioning it as a general framework for the biologically informed modeling of spatially resolved molecular data.

Integration of transcriptional regulatory topology. In addition to protein–protein interactions, gene expression is also governed by transcriptional regulatory relationships, in which one gene directly influences the transcriptional activity of another. Unlike PPI networks, transcriptional regulatory networks are inherently directed and may be signed, since a regulator can either activate or repress its target. In the following we suggest a possible strategy to incorporate this information. We define a signed directed regulatory matrix

$$T \in \mathbb{R}^{K_t \times K_t},$$

where K_t denotes the number of genes retained in the transcriptional regulatory network after filtering, and each entry T_{ij} represents the regulatory effect of gene i on gene j. Specifically, $T_{ij} > 0$ indicates activation, $T_{ij} < 0$ indicates repression, and $T_{ij} = 0$ indicates the absence of a known regulatory interaction. The magnitude $|T_{ij}|$ reflects the confidence or strength of the regulatory interaction.

Let $c_i \in \mathbb{R}^R$ denote the latent representation of gene i, corresponding to the ith row of the gene factor matrix $C \in \mathbb{R}^{K \times R}$. To enforce consistency between the learned gene embeddings and the signed directed transcriptional regulatory structure, we define the following regularization term:

$$R_{\text{TRN}} = \frac{1}{|\mathcal{E}_{\text{TRN}}|} \sum_{(i,j) \in \mathcal{E}_{\text{TRN}}} |T_{ij}| \, \|c_i - \operatorname{sgn}(T_{ij}) \, c_j\|_2^2 \,,$$

where

$$\mathcal{E}_{\text{TRN}} = \{(i, j) \mid T_{ij} \neq 0\}$$

denotes the set of directed regulatory interactions. Under this formulation, activating interactions encourage similar latent representations between regulator and target, whereas repressing interactions encourage opposite latent representations. Minimizing R_{TRN} therefore promotes transcriptionally coherent gene embeddings that are consistent with known signed regulatory dependencies.
